# Non-cytopathic herpes simplex virus type-1 isolated from acyclovir-treated patients with recurrent infections

**DOI:** 10.1038/s41598-022-05188-w

**Published:** 2022-01-25

**Authors:** Subrata Roy, Soumi Sukla, Abhishek De, Subhajit Biswas

**Affiliations:** 1 Infectious Diseases and Immunology Division, CSIR- Indian Institute of Chemical Biology, 4, Raja S.C. Mullick Road, Kolkata, West Bengal 700032 India; 2 Department of Pharmacology and Toxicology, National Institute of Pharmaceuticals Education and Research, 168, Maniktala Main Road, Kolkata, West Bengal India; 3 Department of Dermatology, Calcutta National Medical College and Hospital, Kolkata, West Bengal India; 4 Academy of Scientific and Innovative Research (AcSIR), Ghaziabad, Uttar Pradesh India

**Keywords:** Microbiology, Molecular biology, Diseases, Pathogenesis

## Abstract

Herpes simplex virus (HSV) usually produces cytopathic effect (CPE) within 24-72 h post-infection (P.I.). Clinical isolates from recurrent HSV infections in patients on Acyclovir therapy were collected between 2016 and 2019 and tested in cell cultures for cytopathic effects and further in-depth characterization. Fourteen such isolates did not show any CPE in A549 or Vero cell lines even at 120 h P.I. However, these cultures remained positive for HSV-DNA after several passages. Sequence analysis revealed that the non-CPE isolates were all HSV-1. Analysis of the thymidine kinase gene from the isolates revealed several previously reported and two novel ACV-resistant mutations. Immunofluorescence and Western blot data revealed a low-level expression of the immediate early protein, ICP4. Late proteins like ICP5 or capsid protein, VP16 were almost undetectable in these isolates. AFM imaging revealed that the non-CPE viruses had structural deformities compared to wild-type HSV-1. Our findings suggest that these strains are manifesting an unusual phenomenon of being non-CPE herpesviruses with low level of virus protein expressions over several passages. Probably these HSV-1 isolates are evolving towards a more “cryptic” form to establish chronic infection in the host thereby unraveling yet another strategy of herpesviruses to evade the host immune system.

## Introduction

Herpes simplex virus (HSV) infections commonly causing cold sores and genital herpes continue to be a major public health concern with more than 90% of the global population being infected with it. HSV-1 spreads through oral contacts and mainly causes cold sores, whereas HSV-2 is sexually transmitted and primarily responsible for genital herpes^1^. An estimated 492 million people are living with HSV-2 infection, equivalent to 13.2% of the world’s population aged 15–49 years. About 3752 million people had HSV-1 infection at any site, equivalent to a global prevalence of 66.6% in 0–49 years old individuals in the year, 2016^2^.

The global burden of HSV-1 causing labial infection is huge and HSV-1 causing genital herpes is also substantial^3^. Most of the HSV-1 or HSV-2 infected persons remain asymptomatic and harbour the infection lifelong. HSV is neurotropic and establishes latency in the neurons^4^. Herpes simplex viruses cause ulcerative lesions in patients which are painful. Furthermore, genital herpes plays a potential role in the acquisition and transmission of the human immunodeficiency virus^5^. Acyclovir is the “drug of choice” and administered orally (as such or as a prodrug) to treat the herpes lesions^6,7^.

The growth characteristics and pathogenicity of HSV in people, with varied immune status, especially in developing and under-developed parts of the world, are poorly understood. Some HSV strains can cause overt diseases, whereas others are less virulent but reactivate from time to time despite suppressive or prophylactic therapy^8^. Different patterns of growth characteristics had been observed when HSV clinical isolates were attempted to be grown and isolated in cell cultures^9^. Several studies have shown that the γ_1_ 34.5 protein is essential for HSV pathogenesis in animal models. Deletion mutations in γ_1_ 34.5 protein blocked viral egress^10^. Again, certain spontaneously occurring HSV variants from clinical isolates, selected to novel helicase-primase inhibitors, showed altered growth characteristics (in cell culture) and pathogenicity (in animal models)^11^. Molecular studies have established that single mutations in HSV-1 UL5 helicase (e.g. K356Q, G352R and N342K) alone are often sufficient for the observed phenotypes in case of these variants^12,13^. However, all the aforesaid studies involved HSV clinical or laboratory strains that are cytopathic in nature and produced visible plaques in permissive cell lines.

In Kolkata, a densely-populated metropolitan city in Eastern India, many patients in a Hospital Outdoor Clinic had been reporting painful recurrent herpes infections which were non-responsive to repeated Acyclovir treatment regimes. Oral or genital swabs from eighteen such patients were collected and a detailed study was carried out to characterize these viruses.

## Results

### Cytopathic and non-cytopathic clinical herpes samples

**Figure 1:**
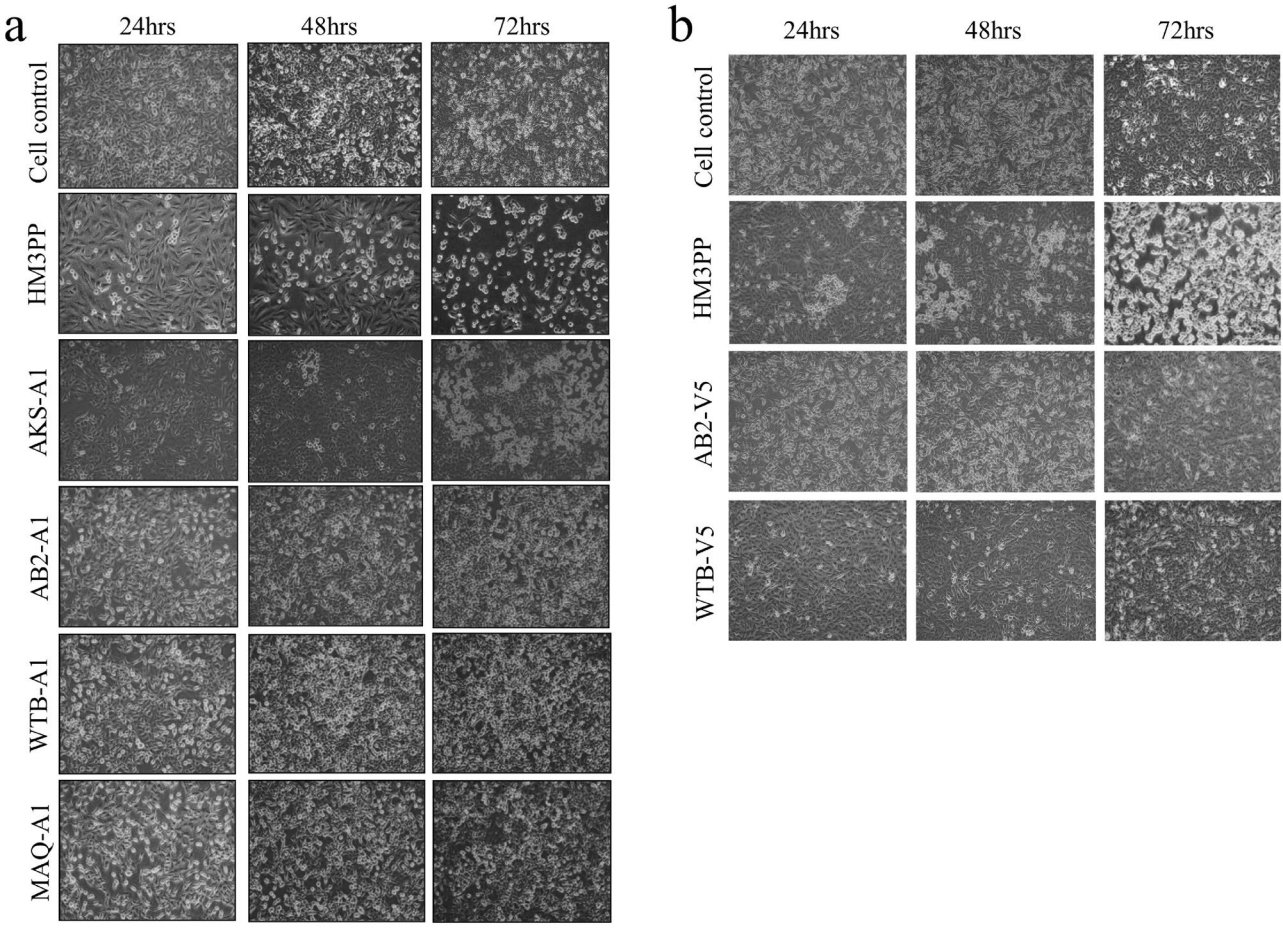
Representative images of infection of HSV clinical isolates in cell culture. (**a**) Course of HSV infection in A549 cells up to 72 h P.I. Images were taken at 200X magnification. Clinical isolates like HM3PP (HSV-1 wt), AKS-A1 (HSV-2 wt) were cytopathic and showed classical plaque formation over time, albeit to different degrees-AKS-A1 being slower compared to HM3PP. In contrast, HSV-1 clinical isolates like AB, WTB and MAQ did not show any distinguishable change compared to uninfected cells over the same time period and were, therefore, non-cytopathic. “A” represents A549 cells and the adjacent number denotes the passage number. (**b**) Non-cytopathic HSV-1 isolates (primarily cultured in A549 cells), showed no signs of HSV-specific cytopathic effect in Vero cells up to 72 h P.I. The same feature held true even at 120 h P.I. (Supplementary Fig. S1). “V” represents Vero cells and the adjacent number denotes the passage number. (Corresponding images of the isolates at 96 h and 120 h P.I. have been shown in Supplementary Fig. S1).

Oral or genital swabs were collected from all patients under study (Table 1). After initial processing, the filtered materials were inoculated in A549 cell culture (lung epithelium cell line). Four of the eighteen isolates namely HM3PP, PB3PP, AKS-A1 and BKN-A1 showed visible cytopathic effect (CPE) which was evident from the classical plaque formation within 48–72 h (Fig. 1a). However, the other fourteen isolates namely AB2, WTB, MAQ and so on didn’t show any CPE in 48–72 h (Fig. 1a). They were then cultured up to 120 h and checked for signs of any slow-growing HSV, which might show CPE later. However, no CPE was observed in case of these isolates even at five days P.I. These isolates were termed as non-CPE samples. The A549 primary yields of all these viruses were subjected to multiple blind passages. No CPE was observed even after five such passages in A549 cells.

**Table 1:** Details of HSV clinical isolates.

Isolate No	Patient Age/Sex	Clinical context*	Sample Name	HSV Type	CPE in A549 (Lung epithelium)	CPE in Vero (kidney)	Nucleic Acid	Replication pattern
1	42 M	Labial	HM3PP	HSV-1	+	+	+	Normal
2	40 M	Labial	PB3PP	HSV-1	+	+	+	Normal
3	32 M	Genital	AB2	HSV-1	-	-	+	Slow
4	32 F	Genital	WTB	HSV-1	-	-	+	Slow
5	18 F	Labial	MAQ	HSV-1	-	-	+	Slow
6	30 F	Genital	RBG	HSV-1	-	-	+	Slow
4	23 M	Labial	AM	HSV-1	-	-	+	Slow
8	18 F	Genital	TH	HSV-1	-	-	+	Slow
9	24 F	Labial	SS	HSV-1	-	-	+	Slow
10	24 M	Genital	ASW	HSV-1	-	-	+	Slow
11	48 M	Genital	SKP	HSV-1	-	-	+	Slow
12	33 M	Labial	SBB	HSV-1	-	-	+	Slow
13	38 M	Genital	SHL	HSV-1	-	-	+	Slow
14	38 M	Genital	PML	HSV-1	-	-	+	Slow
15	42 M	Genital	RSW	HSV-1	-	-	+	Slow
16	20 F	Oral	MBB	HSV-1	-	-	+	Slow
17	39 M	Genital	AKS-A1	HSV-2	+	+	+	Slower than HSV-1 (HM3PP)
18	67 M	Genital	BKN-A1	HSV-2	+	+	+	Normal

All the patients had recurrent HSV infection and received Acyclovir treatment, except PB; their age is shown in years. “A” represents A549 cells and the adjacent number denotes the passage number.

The primary A549 yields of the non-CPE samples were also passaged in Vero cells (kidney cell line) to check for the growth of the viruses in an alternate cell line commonly used for HSV culture. These samples again didn’t show any CPE while similar A549 yields of the cytopathic viruses showed plaque formation in Vero cells within 48-72 h. In the Vero cells also, the yields of the non-CPE samples were blindly passaged five times but no signs of CPE were observed (Fig. 1b; Supplementary Fig. S1).

### Genetic evidence for the presence of HSV

PCRs targeting partial HSV-1 *UL5* helicase and full-length thymidine kinase (tk) genes (primer details in Table 2) were performed on DNA extracted from total yields of the infected A549 or Vero cells. The results confirmed the presence of the HSV DNA for both cytopathic and non-CPE viruses (Supplementary Figs. S2–S4). However, it is noteworthy that the PCR amplified products were fainter for non-CPE viruses compared to the CPE viruses. Supernatants from the virus infected cell cultures were also checked, but no evidence of the presence of HSV-1 DNA was found in case of the non-CPE isolates, within the level of the PCR-based detection.

**Table 2:** Primers used in PCRs and for DNA sequencing for *UL5* and tk gene.

Primer name	Sequence (5ʹ–3ʹ)	Product size (bp)	Remarks	References
*UL5*-4F	TTTACAAAGCTGTCGTCACG	302	Amplification of *UL5* gene	^14^
*UL5*-4R	TGTCGGTCAAGGAGTTTGAC			
H1TK-F1	TTTTATTCTGTCTTTTTATTGCCGTCA	1386	Amplification of tk gene	^15^
BTK-12R	CGAATTCGAACACGCAGAT			
H1TK-CF	CCAAGCTTCATGGCTTCGTACCCCTGCCATCAACACG	1144	Amplification and sequencing of tk gene	This work
H1TK-CR	GGAATTCTCAGTTAGCCTCCCCCATCTCC			
H1TK-F3	ACGATGTTTGTGCCGGGCAAGGTC		Sequencing of tk gene	^15^
H1TK-R2	CATCGCCGCCCTCCTGTGCTACCC			
E225K-F	CAGCGCCCCGGCAAGCGGCTTGACC		Forward SDM Primer	This work
E225K-R	GGTCAAGCCGCTTGCCGGGGCGCTG		Reverse SDM Primer	This work
R212K-F	CTTCCGGAGGACAAACACATCGACCG		Forward SDM Primer	This work
R212K-R	CGGTCGATGTGTTTGTCCTCCGGAAG		Reverse SDM Primer	This work

It was observed that the primary A549 cell yields for the non-CPE viruses after blind passages in A549 or Vero cells still remained positive for viral DNA by PCR (albeit faint bands). After blind passages, yields were subjected to the *UL5*/tk PCR and the corresponding bands in the agarose gel electrophoresis indicated the presence of HSV for cytopathic as well as non-CPE viruses (Supplementary Figs. S2, S4). As mentioned before, no plaques were visible in the infected cells in course of these passages.

Bi-directional sequencing of PCR products of HSV tk gene (Supplementary Figs. S3–S4) and subsequent analysis revealed that AKS-A1 and BKN-A1 were HSV-2 while the rest of the isolates were HSV-1 (HM3PP, PB3PP, AB2, WTB, MAQ and so on) (Supplementary Fig. S5). Hence all the fourteen non-CPE viruses reported in this study belonged to HSV-1. Among the cytopathic viruses, two were HSV-2 (AKS-A1 and BKN-A1) and two were HSV-1 (HM3PP and PB3PP). Except PB3PP, these cytopathic HSV were from patients reporting recurrent infections although they had received ACV therapy.

Amino acid sequence analysis of the tk gene revealed several mutations in case of the non-CPE viruses with respect to the reference tk sequences (Table 3). Some noteworthy mutations were A147G, D162N, V191G and R212K in AB2 and E225K in case of AB2, WTB and AM. The isolate RBG had two changes, G56V and Q89R. MAQ showed a change in the TK amino acid sequence (G56A); in addition, a frameshift mutation was also observed due to deletion of a cytosine residue at the 900th position of the tk gene. Consequently, the ORF of MAQ TK could not be determined due to lack of stop codon and therefore, MAQ TK has been predicted as non-functional. The two cytopathic HSV-1 isolates, namely HM3PP and PB3PP showed only one polymorphism (G21V) compared to the reference HSV-1 tk wild type (wt) sequences (Supplementary Fig. S5a).

**Table 3:** Polymorphisms/mutations observed in the HSV-1 TK.

HSV Type	Serial number	Polymorphism/Mutation	Found in sample	Domain	Previously reported mutation	Function	Sensitivity/resistance to ACV	References
HSV-1 isolates	1	G21V	PB3PP, HM3PP	Non-conserved	Not reported	ACV sensitive	Not applicable (NA)	This study
	2	G56V	RBG-V2	ATP-binding site	G56S	ACV resistant	IC50 1.9 µM: 11-fold	^16^
	3	G56A	MAQ-V1, MAQ-A1	ATP-binding site	G56S	ACV resistant	IC50 1.9 µM: 11-fold	^16^
	4	Q89R	RBG-V2	Non-conserved	Q89R	Natural polymorphism	NA	^17^
	5	A147G	AB2-A2, AB2-V1	Non-conserved	Not reported	Unknown	Not done	^18^
	6	D162N	AB2-A2, AB2-V1	Conserved region (nt binding site)	D162N	ACV resistant	IC50 11.0 µM: 64-fold	^19,20^
	7	V191G	AB2-A2, AB2-V1	Non-conserved	V191A	Spontaneous mutation	NA	^18^
	8	R212K	AB2-A2, AB2-V1	Non-conserved	Not reported	ACV resistant	70% resistance to ACV at 4.4 µM (> IC90)	This study
	9	E225K	AM-V2, WTB-V2, WTB-A2, AB2-A2, AB2-V1	Non-conserved	Not reported	ACV resistant	60% resistance to ACV at 4.4 µM (> IC90)	This study
	10	S263L	WTB-V2, WTB-A2	Non-conserved	S263L	Natural Polymorphism	NA	^21^
	11	P300Del	MAQ-V1, MAQ-A1	Non-conserved	Not reported	ACV resistant	Fully-resistant, IC50 > 44 µM	This study
HSV-2 isolates	12	G39E	AKS-A1, BKN-A1	Non-conserved	G39E	Natural polymorphism	NA	^22^
	13	N78D	AKS-A1	Non-conserved	N78D	Natural polymorphism	NA	^23^
	14	L263P	AKS-A1	Non-conserved	Not reported	Spontaneous mutation	NA	This study

### Analysis of growth characteristics

**Figure 2:**
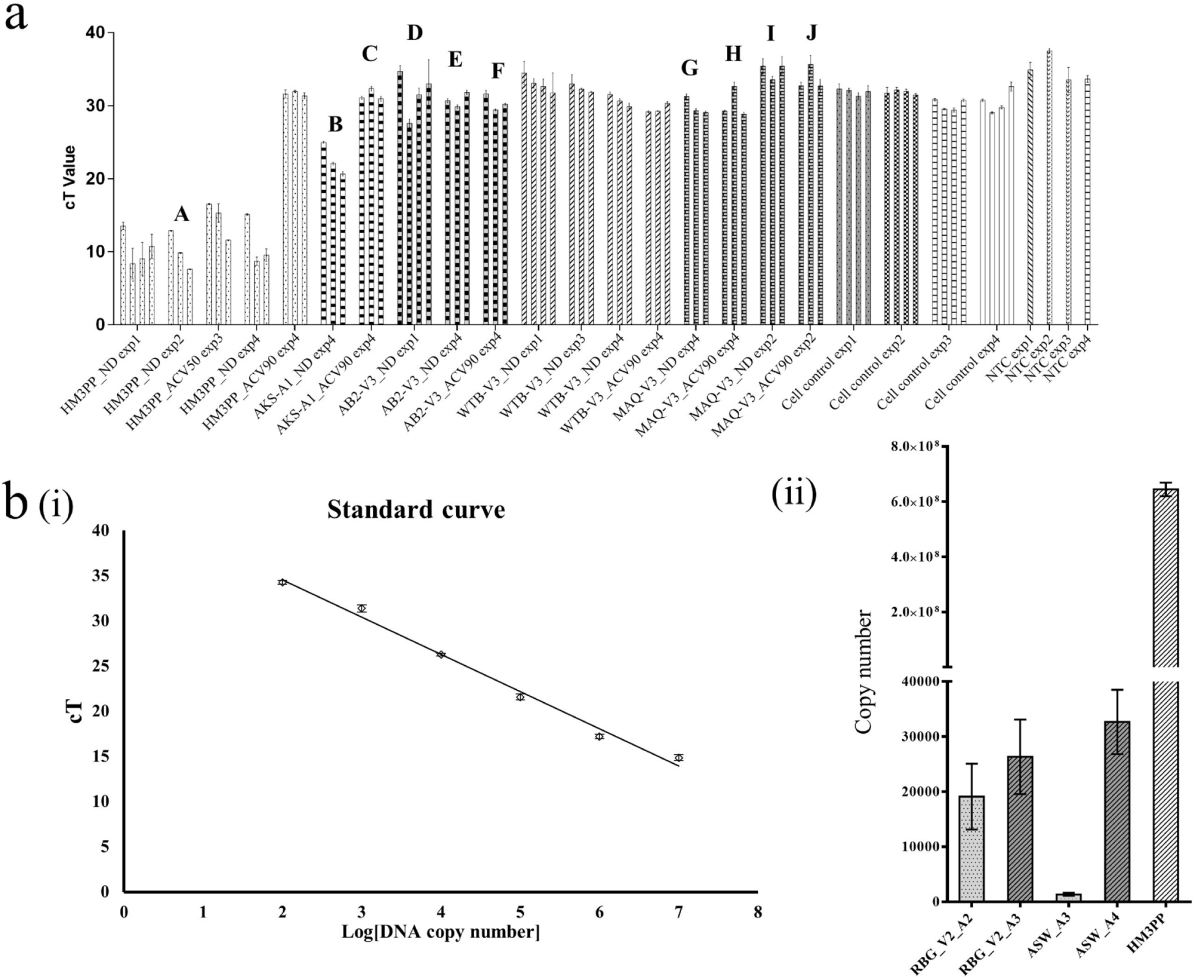
Comparison between cytopathic and non-cytopathic virus replication over time in cell culture by qPCR. (**a**) The graph represents cT values of the isolates at different time points. Six-well plates, seeded with Vero or A549 cells, were infected with, 200 µl virus-infected cell lysate for non-CPE viruses. In case of the cytopathic viruses, cell monolayer was inoculated with 50 p.f.u. virus/well. Replicate cell cultures were also treated with IC50 (experiment 3) or IC90 (experiment 2 and 4) concentrations of ACV. “ND” denotes “no-drug control”. Infected cells were harvested at different time points and total DNA was isolated from the harvested samples. Maximum amount of DNA permissible (160 ng) was subjected to qPCR for each sample. The columns corresponding to each condition (marked on the X-axis) represent the mean cT value (± SD) at 48 h, 72 h, 96 h, and 120 h P.I. respectively, starting with cT value at 48 h P.I. In case of the cytopathic viruses, the maximum virus DNA copies could be detected at 72 h P.I. Thereafter, the number of uninfected cells declined resulting in a decrease in the virus yield as well. This pattern could be observed in non-CPE viruses like MAQ and AB2, in low amount. On the other hand, WTB showed a significantly slow replication pattern. NTC represents the background amplification observed for the no-template control. The qPCR products were checked in 1% agarose gel electrophoresis to confirm the amplification. Supplementary Fig. S6 i-iii represent gel images of representative qPCR experiments demarcated by small letter alphabets shown on the top of the panels. (b) (i) cT values were proportional to the DNA copy numbers as measured by the dilution of known copy number of the plasmid containing the target sequence. This representative data set provides a standard curve of cT values, from which copy numbers of the HSV-1 isolates were calculated. b (ii) DNA from cytopathic and non-CPE virus-infected Vero or A549 cells were checked by qPCR and copy number from total cells per well, was determined. Increment in the number of HSV-1 DNA copy number could be observed over multiple passages (Supplementary Fig. S6 iv). “A” and “V” represent A549 and Vero cells respectively and the adjacent numerical denotes the cell passage number.

Infected cells (primary as well as secondary passages) from both cell lines (with and without ACV-treatment) were collected at different time points and total cellular yield was checked for growth characteristics using real-time PCR analysis. Although the same amount of total DNA was used for each sample, non-CPE HSV-1 showed slower and lower levels of replication pattern compared to the cytopathic viruses like HM3PP and AKS-A1 as evident from higher cT values (Fig. 2a).

In Vero cultures, for certain non-CPE isolates like AB2, maximum amount of viral DNA levels was detectable at 48-72 h P.I. and became undetectable then onwards, as observed up to 120 h P.I. samples (Fig. 2a). For other non-CPE viruses like WTB and MAQ, low and similar levels of DNA remained detectable up to 96 h P.I. (Fig. 2a).

Successful PCR amplification was confirmed by observation of visible bands in agarose gel electrophoresis (AGEP) of the qPCR samples after completion of the real-time run (Supplementary Fig. S6). From a series of qPCR experiments, it was evident that AKS-A1, the HSV-2 isolate showed slower growth and somewhat less sensitivity to ACV compared to HSV-1 HM3PP (Fig. 2a).

A two-times cell passaged (A549) non-CPE virus, AM-A2 when inoculated at 300 µl in 10^5^ Vero cells resulted in no CPE at 48 h P.I. and the total virus yield was estimated at 300 copies of HSV-1 by qPCR.

Sonicated cell lysates of non-CPE HSV-1, ASW and RBG (four times and five times cell-passaged respectively) when inoculated in sub-confluent cultures of A549 cells in 12-well plates (2 × 10^4^ cells/well) resulted in approximately 3.0 × 10^4^ copies HSV-1 at confluency (i.e., 4 × 10^5^ cells/well) at 96 h P.I. (Fig. 2b). In contrast, 20 p.f.u. HSV-1 wt, (HM3PP) inoculated into a similar monolayer produced a yield of approximately 6.0 × 10^8^ copies by 48 h P.I. (Fig. 2b).

### Expression study of an early and a late HSV-1 protein

**Figure 3:**
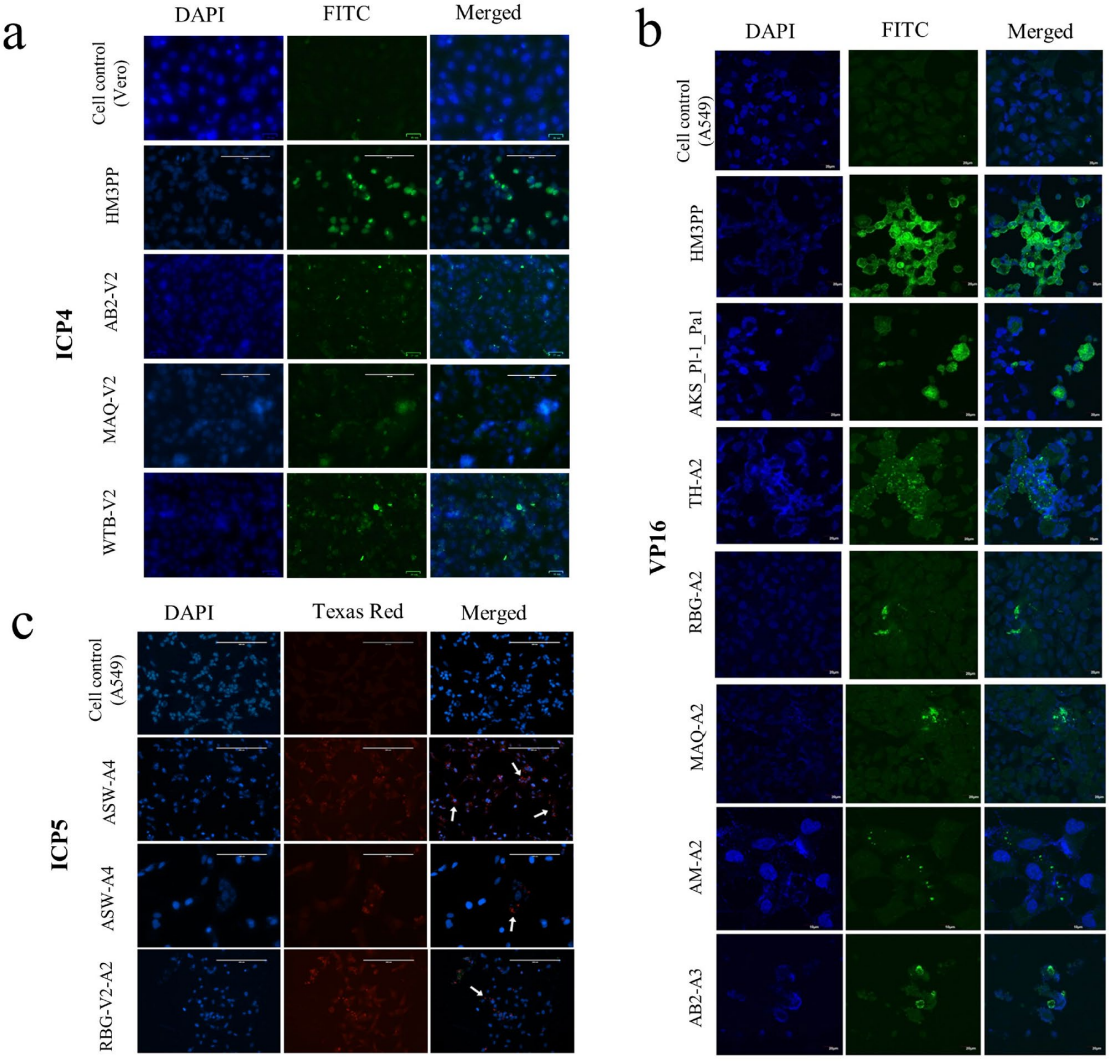
Representative immunofluorescence images showing difference in expression pattern of early and late proteins in case of non-CPE clinical HSV-1 isolates in different cell lines. (**a**) Images of Vero cells infected with cytopathic and non-cytopathic HSV-1 isolates for ICP4 detection. Cells were stained for HSV-early protein ICP4 using anti-ICP4 Mab, tagged with Alexafluor 488. Low Expression of the early protein surrounding the nucleus could be observed in case of non-CPE isolates like AB2-V2, MAQ-V2 or WTB-V2 compared to cytopathic HSV-1 (HM3PP). (**b**) Immunofluorescence staining of HSV-1-infected A549 cells for VP16 late protein. Compared to cytopathic viruses very low amount of expression can be spotted in the cytoplasm in case of non-CPE viruses after 72 h P.I. on staining with anti-VP16 Mab, tagged with Alexafluor 488. Compared to the extent of early protein expression, the non-CPE viruses (i.e., MAQ-A2, AM-A2 etc.), showed discrete/localized distribution of VP16 expression. (**c**) Immunofluorescence images of non-cytopathic virus-infected A549 cells for ICP5. Cells were stained with Alexafluor 568 tagged anti-HSV-ICP5 (HSV capsid protein) antibody and checked by immunofluorescence microscopy. Compared to cell control, localized expression of ICP5 (white arrow) can be observed for the non-CPE viruses after 72 h P.I. without showing any cytopathic signs of HSV-1 infection. Row 3 presents a magnified view of ASW-A4 for better visualization. (**a**–**c**) Cell control denotes uninfected cells.

Immunofluorescence results further supported the observed low-level virus replication for the non-CPE viruses. HSV-1 immediate-early protein, ICP4 and late protein, VP16 were viewed in infected cells, using Alexafluor 488-tagged monoclonal antibody against the respective proteins (Fig. 3a). ICP4 staining showed low level but scattered expression across the cell monolayer. When stained for the late structural protein, VP16 or ICP5, low level but focal staining of A549 cells was observed for the non-CPE viruses compared to the cytopathic viruses and the uninfected control (Fig. 3b,c). Singled out cells expressing VP16 or ICP5 were observed and surrounding cells appeared to be uninfected in contrast to more scattered staining of a much higher number of cells when stained for ICP4.

**Figure 4:**
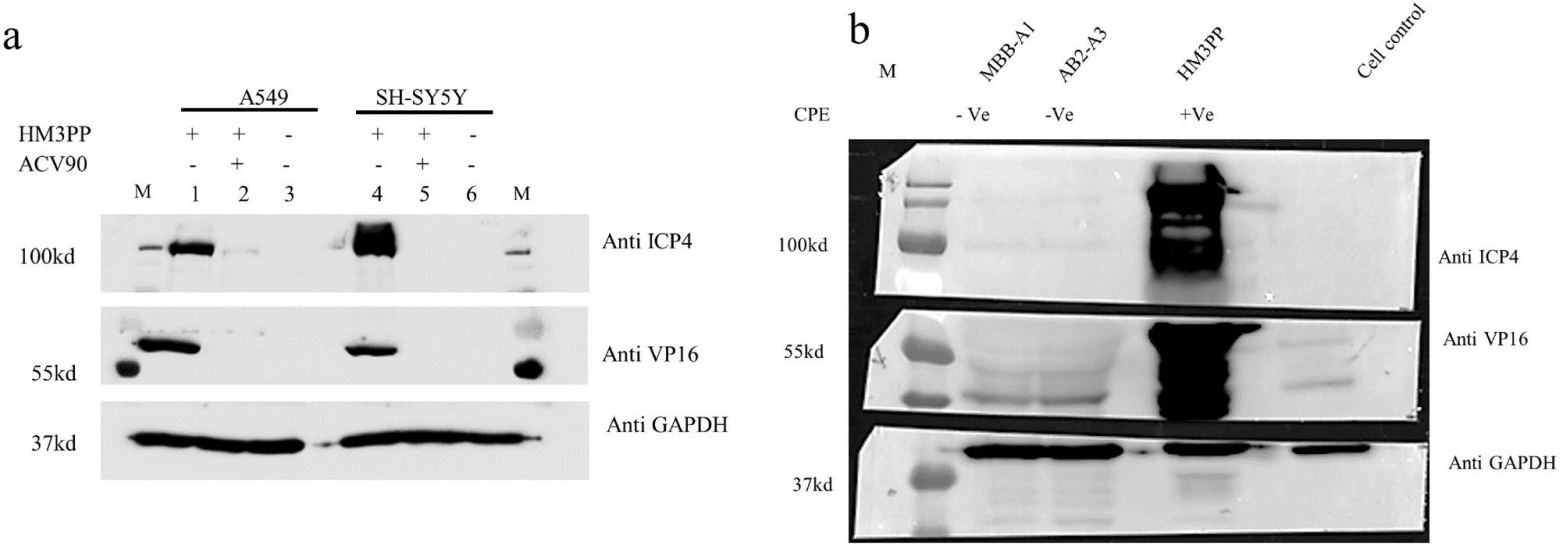
Expression of immediate early protein (ICP4) and late protein (VP16) in different cell lines for HSV-1 wt and two representative non-CPE viruses (MBB-A1 and AB2-A3). (**a**) Epithelial (A549) and neuronal (SH-SY5Y) cell lines were infected with 50p.f.u. HM3PP for 48 h. Yields from virus-infected cells had been marked “ + ” and those from non-infected control as “-” (first row). Incubation was done with ( +) or without (-) ACV (4.4 µM, > IC90 for HM3PP) (second row). 40 ng of the cellular yields were separated on 10% polyacrylamide gels; transferred to a nitrocellulose membrane and detected by probing the membrane for ICP4 and VP16. (**b**) A549 cells, infected with non-CPE virus isolates were concentrated with 100 kD filter (Amicon) before running on the gel. Very low amount of IE protein expression was observed in case of non-CPE viruses. Even at high exposure, very faint bands were noticed for these isolates for each target protein. Late gene expression such as VP16 was not detectable for the non-CPE isolates. Original Western blot images have been presented in the Supplementary Figs. S7 and S8.

These findings were also established by Western blot analysis. When HSV-1 infected cell lysates were checked against early protein ICP4 and capsid protein VP16, low amount of ICP4 protein was detected in the case of non-CPE viruses compared to the cytopathic viruses. The expression of VP16 was even more negligible (Fig. 4a,b). Similarly, ICP5, another capsid protein expressed lately during HSV-1 replication, was also hardly visible in WB (data not shown).

### Sensitivity to Acyclovir

The sensitivity of the plaque-forming HSV-1 and 2 isolates to ACV was tested by plaque reduction assay (PRA) in Vero cells. HSV-1 isolates like HM3PP and PB3PP showed IC50 of 0.2 ± 0.08 µM (SD) and 0.06 ± 0.03 µM respectively. The IC90 for these HSV-1 wt isolates was approximately 0.4 ± 0.04 µM. On the other hand, HSV-2 isolates AKS-A1 and BKN-A1 showed an IC50 of 1.3 ± 0.02 µM and 2.7 µM respectively. All cytopathic HSV were found responding to ACV in cell culture, however, the HSV-2 isolate BKN-A1 was about 14-fold less sensitive compared to the HSV-1 isolate, HM3PP. The ACV-susceptibility of these plaque-forming clinical isolates fell within the typical range of IC50 values for HSV in cell culture, observed between 0.09–4.0 µM for HSV-1 and 0.13–9.7 µM for HSV-2^24^. Again, the HSV-2 isolate AKS-A1 seemed to have slower growth properties compared to the cytopathic HSV-1 isolate, HM3PP (Fig. 2a). AKS-A1 and BKN-A1 showed some amino acid polymorphisms in the TK (Table 3; Supplementary Fig. S5b) but both were susceptible to ACV as evident from their IC50 values.

In order to check ACV-sensitivity/resistance of the non-CPE clinical isolates, the full-length tk gene PCR products (e.g. MAQ) were transferred (by means of homologous recombination) to plaque forming HSV-1 wt (e.g., HM3PP). Successful transfer of the MAQ-tk gene was confirmed by visualization of plaques at higher-than-expected frequency (approximately 10 from 10^4^ p.f.u. inoculum) in an inhibitory concentration of ACV (4.4 µM; > IC90 for HSV-1). Such resistant plaques were collected and expanded for further analysis. For MAQ, the resultant recombinant virus (HM-MAQ tk-rec) was found to be fully resistant to ACV (IC50 > 44 µM). DNA sequence analysis of such recombinant virus showed successful transfer of MAQ-tk to target HSV-1 wt, which was otherwise ACV-sensitive. The tk gene from HM-MAQ tk-rec showed the G56A mutation and the same novel deletion of the last C nt at 900th nt position in a homopolymeric stretch of five C residues as was originally observed in MAQ tk gene (Supplementary Fig. S5a). This led to ORF shift and lack of stop codon for the TK protein, resulting in a TK null virus. Therefore, MAQ is a TK null non-CPE HSV-1 isolate, fully resistant to ACV.

Two other mutations, R212K and E225K were introduced in the backbone of HM3PP tk gene cloned into pcDNA-EGFP 3.1 plasmid by SDM. These recombinant plasmids were transfected in A549 to supply the mutant TK enzyme *in trans* in cells, super-infected with HM-MAQ tk-rec (TK null virus) and topped with 4.0 µM ACV. Transfection of the wt tk clone revealed that 4.0 µM ACV was effective against both HM3PP and HM-MAQ tk-rec. On the other hand, transfection of the recombinant plasmids containing R212K or E225K mutation showed 70% and 60% resistance towards ACV in case of HM-MAQ tk-rec (Supplementary Fig. S9). In presence of R212K and E225K recombinant plasmids, the TK null virus produced 30% and 40% plaques compared to the no drug controls.

### Visualization of Non-cytopathic viruses by means of Atomic Force Microscopy (AFM)

**Figure 5:**
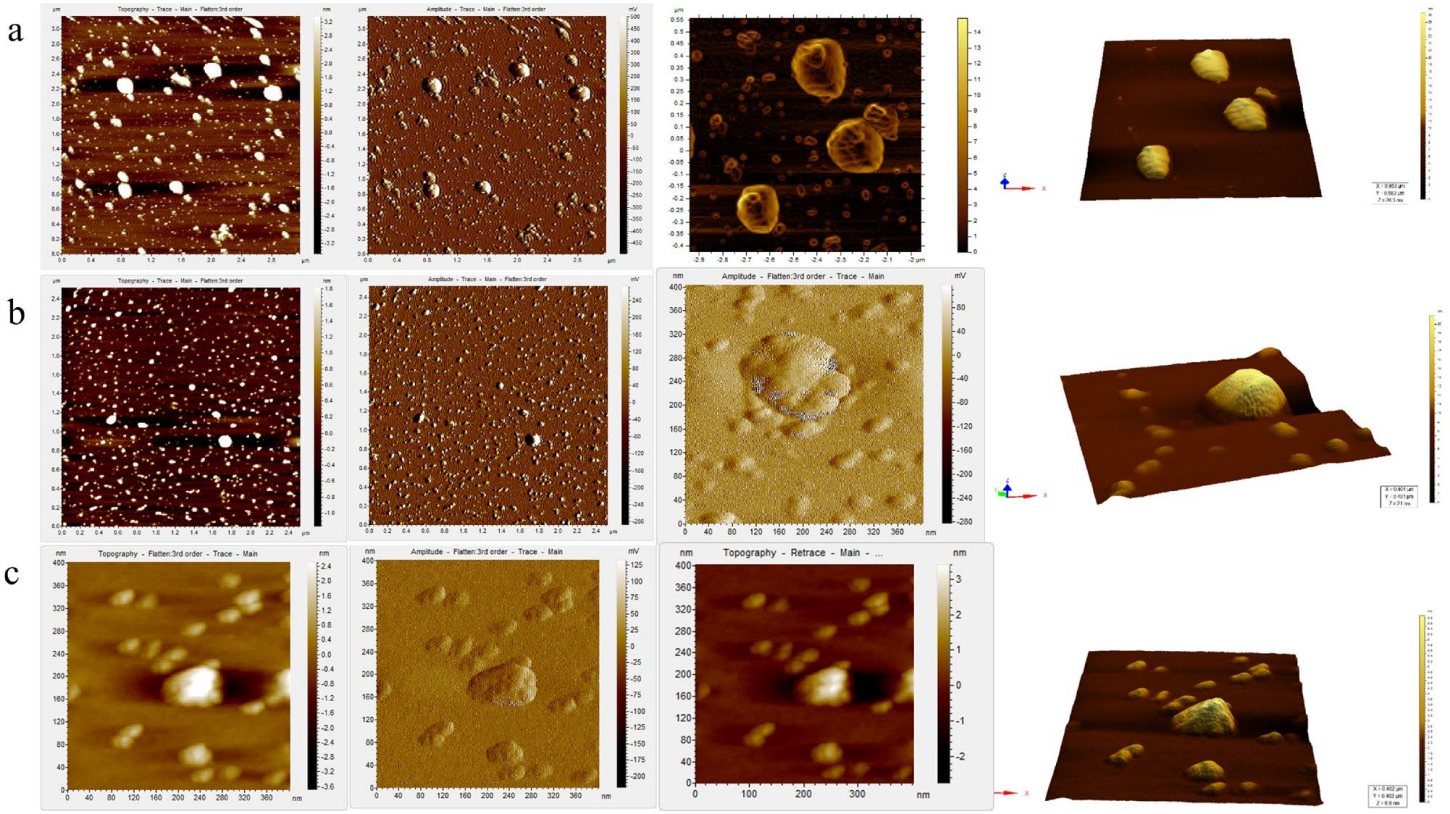
AFM images of the cytopathic and non-cytopathic HSV-1 isolates. (**a**) HM3PP, a representative cytopathic HSV-1 isolate. (**b**) RBG, a representative non-cytopathic HSV-1 isolate: Only a few particles could be seen in a selective field, possibly due to low abundance. (**c**) RBG after immune-concentration with magnetic beads (conjugated with HSV MAb against VP16). Despite washings following immune-concentration, smaller structures could be observed which were possibly the fragments of virus particles.

AFM was carried out for several non-CPE viruses to confirm the presence of intact whole virus particle by visualization. In addition, AFM of HSV-1 wt virus was also accomplished to visualize the original virus structure (positive control). AFM of uninfected cell control was also done to identify and eliminate background noise during imaging. Treatment of 0.2% TritonX 100 removed the lipid envelope from the virus particles and the icosahedral capsid of HSV was exposed. In the case of HM3PP, particles ranging from 100-125 nm were clearly visible (Fig. 5a). A honeycomb pattern of the capsid can be observed when these individual particles were observed at higher magnification (Fig. 5a, panel 3).

On the other hand, due to lower abundance of non-CPE virus (e.g., RBG), none or very few particles ranging from 100-125 nm in size were visible in a particular field (Fig. 5b), even in case of the immuno-precipitated sample (Fig. 5c). Interestingly, repeated imaging confirmed that these particles also showed honeycomb pattern of the capsid upon magnification but the outline of such structures appeared distorted compared to the cytopathic virus particles (Fig. 5b,c).

## Discussion

Previous surveillance studies have shown that in India, 63% of the population was seropositive for HSV-1 and HSV-2. Among them, the prevalence of HSV-1 and HSV-2 was 33.3% and 16.6% respectively. This study corroborates with the previous reports and confirmed that HSV-1 was more prevalent than HSV-2 among the isolates screened^25^.

Patients in an Outdoor Clinic at CNMC Hospital, Kolkata presented with oral and genital herpes, as diagnosed from clinical symptoms and case history. These included fresh cases (like PB3PP) but a great majority were complaining recurrent HSV infections although they were receiving acyclovir treatment. The present study was conducted to characterize these recurrent cases that were refractory to ACV therapy.

For the detection of HSV from clinical swab samples, cell culture-based isolation is the gold standard. HSV can be grown in a number of cell lines. MRC5 and Human Foreskin Fibroblast are often used for HSV culture for their increased susceptibility^26^. A549 is lung carcinoma cell line and HSV replicates well in this cell line. Therefore, we used A549 which was readily available. A549 is an efficient cell line for HSV growth and has been used in other studies as well^27,28^. Filtrates from the swabs from the above-mentioned patients were inoculated in A549 cell line but majority of the samples didn’t show any CPE even when they were cultured for five days or passaged multiple times in a different cell line (Vero). These cell cultures were positive for HSV-specific genes (*UL5*, tk) and HSV-specific proteins (ICP4, ICP5 and VP16), through serial cultures, signifying the presence of the replicating virus.

In general, when permissive cell cultures are infected with HSV in vitro, the virus typically produces CPE as evidenced by rounding of the infected cell; fusion with adjacent cells to form syncytia, and the appearance of nuclear or cytoplasmic inclusion bodies within 24-48hrs^11,29^. However, for slower growing isolates, it takes longer time. CPE and plaque formations are generally considered as a diagnostic marker of HSV infection^30,31^.

Other than a recent report from Mexico^32^, we know of no other precedence of these unusual forms of non-CPE clinical strains of HSV-1. The striking fact is that although the clinical isolates were non-CPE, the patients repeatedly presented with clinical manifestation of HSV infection in the form of oral or genital lesions. Since these patients received ACV therapy on repeated occasions, mutation analysis of the tk gene was carried out and compared with the available database of HSV TK variants/mutants^29^. Some noteworthy mutations were D162N and R212K in AB2 and E225K in case of AB2, WTB and AM.

Among them, D162N had been reported as a drug resistant mutation previously and was found to confer more than 60-fold resistance to ACV^19,20^. R212K and E225K are novel TK mutations identified in this study and estimated to confer 70% and 60% resistance to 4.4 µM ACV respectively. MAQ2 showed a change in the TK amino acid sequence (G56A); in addition, a frameshift mutation was also observed due to deletion of a cytosine residue at 900th position of the tk gene. Consequently, the ORF of MAQ TK could not be determined due to lack of stop codon and therefore, MAQ TK has been predicted as non-functional. The deletion mutation at the 900th nucleotide position of tk gene from MAQ was PCR-amplified and marker-transferred to the wt HSV-1 and it was found to be fully resistant against ACV, with IC50 > 44 µM concentration. The two cytopathic HSV-1 isolates, namely HM3PP and PB3PP showed only one polymorphism (G21V) compared to the reference HSV-1 TK wt sequences. The isolate AB2 was from a male patient who repeatedly reported genital lesions despite ACV therapy. It is not surprising that the TK of AB2 harboured three identified ACV-resistance mutations (D162N, R212K and E225K). There were still other mutations like A147G, V191G in AB2; G56V and Q89R in RBG; G56A in MAQ and S263L in WTB but their exact functional significance was not clearly known and not determined in this study. It is however known from other studies that the G56S mutation conferred 11-fold ACV-resistance (Table 3). Previous in vitro analysis of mutations in ATP-binding site confirmed that the G56V mutation (as in RBG) inactivated the enzyme activity by blocking access to ATP molecules^33^. The mutation, Q89R was found to be sensitive to ACV^17^. Other mutation (R89W) at this position showed resistance to ACV^16^. Another amino acid substitution S263L, observed in WTB, had been previously described as a TK polymorphism^21^.

Immunofluorescence studies revealed that the non-CPE isolates were expressing ICP4, an immediate-early protein, in a scattered manner but visibly at lower levels compared to cytopathic HSV-1. On the other hand, focal or extremely low expressions of the late proteins (i.e., VP16 and ICP5, which are capsid proteins) were visible in the infected cells. Even in the Western blot study, levels of expressions of the late proteins were almost negligible compared to cytopathic viruses. VP16 was previously determined as an important factor for egress and downstream viral assembly^34,35^. These findings indicated that these non-CPE viruses had difficulty in egress from the cells. Previous studies revealed that mutation (s) in γ_1_ 34.5 protein can cause difficulties in virus egress^10^. But reports of such mutation in case of HSV clinical isolates are not available in the database. Further investigation revealed that the growth properties of these non-CPE viruses were slower than the cytopathic ones and very few copies were generated after each passage. For instance, the total yield from 4.0 × 10^5^ A549 cells/well of a 12-well plate was estimated to be approximately 10^4^ copies at 96-120 h P.I. for the non-CPE viruses. In contrast, 20 p.f.u. HSV-1 wt (HM3PP) inoculated into a similar monolayer produced a yield of approximately 6.0 × 10^8^ copies by 48 h P.I. These observations were also reflected in AFM study where the non-CPE viruses were found to be less abundant and deformed compared to the HSV-1 wt.

**Figure 6:**
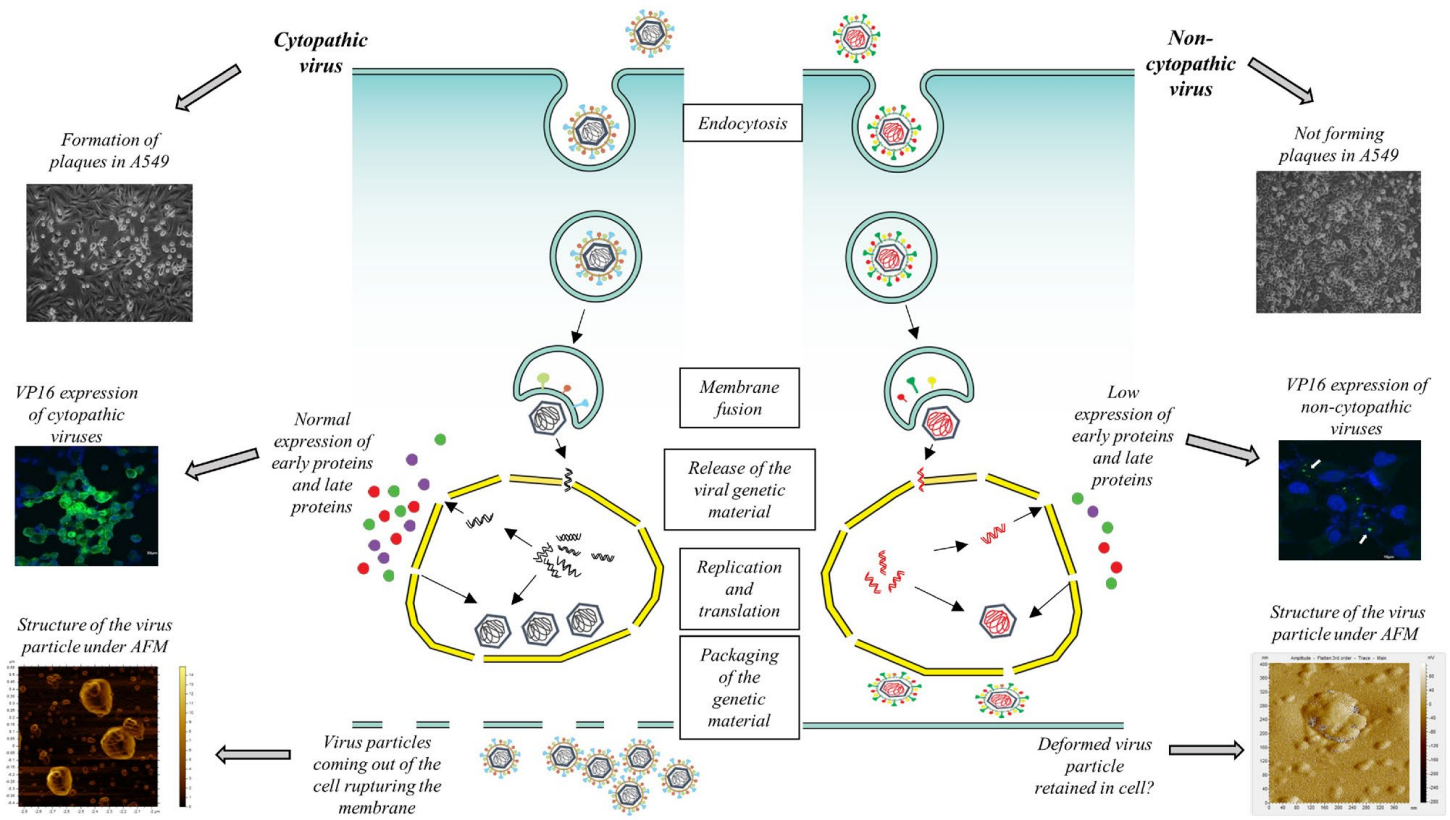
Schematic representation of the difference in pattern of cell infection between cytopathic (left panel) and non-cytopathic HSV-1 isolates (right panel). Normally, HSV-1 enters into the cell through endocytosis and releases nucleic acid into the nucleus where multiple copies of DNA are generated. Viral DNA is transported to cytoplasm for expression of viral proteins, which helps to form capsid particles in the nucleus. After formation of the complete enveloped virus particle, it ruptures the cell membrane and egresses. On the other hand, non-cytopathic virus replication produces lower number of DNA copies resulting in low expression of the virus proteins. Thus, faulty/low level virus replication or packaging hinders proper release of the progeny virions.

As we observed deformities on the surface of the envelope of these viruses, role of glycoproteins also needs to be considered. Viral mutants like gB^−^/gD^−^ had shown accumulation of enveloped virions in the perinuclear region, where the number of extracellular virus particles was reduced^36,37^. On the other hand, gC plays an important role in virus adsorption^38^. Mutation or any aberration in these genes can also lead to the non-CPE feature of these viruses. Nevertheless, these viruses showed recurrent infection/relapse in the patients although they were under ACV therapy. It appears from our analysis that the non-CPE HSV-1 clinical isolates are different from regular HSV-1 cytopathic strains in their capability to cause cytopathic effect in permissive cell lines. They are incapable of characteristic plaque formation, as observed in case of typical HSV-1 strains in 24-48 h P.I. These non-CPE strains replicate really slowly in cells with a very low yield of the virus. Our calculations showed that the 120 h yield was at least 4log10 copies lower compared to 48 h yield from a typical HSV-1 productive infection. This observation was supported by scattered expression of ICP4 similar to wt strain but the level/intensity of expression was lower. In contrast, late or structural protein expression was focal, i.e., only a handful of the initially infected cells produced infectious virus, suggesting a possible defect in egress. AFM data also pointed towards structural deformities in the virions but additional confirmations are required for arriving at a concrete conclusion (Fig. 6).

Studies with HSV laboratory strains have shown that quiescent HSV-1 infection in non-neuronal cells can be achieved when virus was maintained in non-replicative phase. This could happen when virus infections were carried out either with mutant viral strains (mainly with mutations in immediate early genes), in the presence of antiviral drugs, or using special infection conditions, such as high temperature^39^. A recent in vitro study with an engineered laboratory strain, HSV-1 OK22, revealed that abortive infection could cause latency-like state in non-neuronal cell lines spontaneously. Such abortive infection could result in occasional virus reactivation over time. Cells with abortive viral infection failed to produce acute infection and preserved the HSV-1 genome in a dormant state inside the nucleus, bound to cellular histones^40^.

Our findings with real clinical HSV-1 isolates appear similar to the above scenario of abortive infection shown for HSV-1 laboratory strain, but further experimental validation is warranted to really assign abortive infection status to these non-CPE HSV-1 isolates.

One pertinent question is whether partial or damaged viral DNA is passing through cell passages. This question arises from the fact that PCR-positive shedding of HSV is more common than infectious virus shedding^41^. In our case, the isolates were found to be virus DNA-positive even after several passages and cT values showed their increment through successive passages in several instances. So, it is highly unlikely that low level viral nucleic acid present in the original swab inoculum was carried over through five passages and still come PCR/qPCR-positive.

Low levels of viral proteins could be detected in immunofluorescence as well as Western blot experiments. This is another direct proof that the virus was entering into the cells; replicating and expressing viral proteins in “quiescent” state. From all the evidences we could gather so far, it appeared that the virus could infect the cells but there were issues with virus egress, as supported by focal late protein expression and deformed structure in AFM imaging.

Nevertheless, recurrent infection in patients itself indicates the infectious property of these non-CPE isolates. It is to be noted that the samples were collected from these patients when they visited the clinic reporting overt HSV lesions in the labial/genital sites. Recently similar incidents have also been reported from Mexico where clinical HSV isolates from immunocompromised patients were found positive for tk-PCR while one remained negative in in vitro culture^32^. This non-CPE feature is unusual for HSV. Clinical manifestation in HSV-1 infection could be the result of immune reaction of the host. It is possible that in the patients, these defective viruses (with/without ACV-resistance) avoid clearance by low level replication in the cells but the patients show clinical symptoms due to immune response to this sustained virus replication at the site of infection/reactivation.

Overall, these properties might be protecting these viruses from host immune clearance; they show signs of sequestration inside the cells-is it a different form of immune evasion by low-level persistence in infected cells? On top, some of these isolates, indeed, carried one or more ACV-resistant mutations in the TK, which further contributed to their persistence despite ACV therapy.

After decades of extensive use of nucleoside analogues, we are going to face new challenges in HSV management due to the emergence of these “quiescent” strains. The dearth of enough viral DNA and difficulties with in vitro culture precluded us from fully characterising the isolates by means of whole-genome sequencing/reconstruction experiments. We believe that this work would alert the scientific community so that others can investigate further from other perspectives also.

## Methods

### Ethics statement

The present study was approved by the respective Institutional Ethical and Biosafety Committees of CSIR-Indian Institute of Chemical Biology and Calcutta National Medical College (CNMC), Kolkata. Written informed consents (in their native language) were obtained from all the patients/individuals before the sample collection. It is confirmed that all experiments were performed in accordance with relevant guidelines and regulations.

### Study subjects

Eighteen samples were collected from the Calcutta National Medical College (CNMC), Kolkata between 2016 and 2019. Details of the patient information have been documented (Table 1). All the patients were chronic HSV-infected and have a history of receiving Acyclovir treatment except PB3PP, who was diagnosed with HSV-1 for the first time and was not on any antiviral therapy at the time of the collection of samples.

### Cell culture

Two types of epithelial cell lines namely A549 (CCL-185) and Vero (CCL-81) cells and a neuronal cell line SH-SY5Y (CRL-2266) were used in this study. Cell lines were obtained from ATCC and cultured following the standard mammalian tissue culture protocols. Cells were cultured in Dulbecco’s Modified Eagle Medium (DMEM) (SIGMA, UK). Media were supplemented with 10% FBS (GIBCO, USA) for growth (DMEM10) and 1% FBS for the maintenance (DMEM1) of cells. In addition, the media were also supplemented with antibiotics solution (SIGMA, UK) containing 100units/ml penicillin, 100 µg/ml streptomycin and L-glutamine (2 mM).

### Virus culture

Swabs from the infected patients were collected in 400 µl 1XPBS buffer containing penicillin (20 IU/ml), streptomycin (100 µg/ml) and amphotericin B (250 µg/ml). They were filtered using 0.22 µm PES syringe filter (Millipore, Ireland). Approximately, 200 µl of the filtrate was mixed with DMEM1 to make the volume up to 1 ml before inoculating monolayer of A549 or Vero cells in T25 flasks. The culture flask was incubated for 1 h at 37 °C in a CO_2_ incubator for virus adsorption. This was followed by topping the cell monolayer with 4 ml of DMEM (1% FBS).

Plaque forming viruses were titrated and plaques were picked at 72 h (P.I.). Plaques from each cytopathic isolate were subjected to three cycles of plaque-purification and labelled as 3PP (i.e., HM3PP, PB3PP etc.). Stocks were prepared for each isolate for downstream applications.

### DNA extraction and PCR amplification

DNA was extracted from infected cell cultures using the DNeasy Kit (QIAGEN, Germany) following the manufacturer’s instructions. The concentration and purity of the extracted DNAs were checked using spectrophotometry (NanoDrop, Thermo Fisher, USA). DNAs were then subjected to virus-specific PCR using GoTaq 2X Master Mix (Promega, USA). In order to confirm virus infection, HSV-1 *UL5*-4F/4R primers (0.2–0.4 µM) were used (Table 2). A touchdown PCR of 13 cycles with initial heating at 95 °C for 20 s and Ta from 70 °C to 58 °C (diminishing by 1 °C at each cycle) for 20 s followed by an extension at 72 °C for 20 s, was standardised to amplify the *UL5* gene. Rest of the PCR was programmed as follows: initial denaturation at 94 °C (5 min) for 1 cycle followed by 30 cycles of heating at 94 °C (20 s), primer annealing at 58 °C (20 s) and extension at 72 °C (30 s). The final extension was carried out at 72 °C for 1 cycle (10 min).

For amplification of HSV tk gene, two sets of primers were used. First-round amplification was done with H1TKF1 and BTK12R primer set while the nested PCR was performed with the internal set of primers, namely H1TKCF and H1TKCR (Table 2) (Supplementary Fig. S3). Reaction mix for tk amplification was prepared with Accumprime GC rich PCR system (Invitrogen, USA) where forward and reverse primer concentration was 0.2–0.4 µM. A touchdown PCR of 15 cycles with initial heating at 95 °C for 20 s and Ta from 70 °C to 56 °C for 20 s followed by extension at 72 °C for 20 s, was standardised to amplify the tk gene using H1TKF1 and BTK12R primers. The cycling conditions for the remainder of the first-round PCR comprised of initial denaturation at 95 °C (5 min) for 1 cycle followed by 30 cycles of heating at 94 °C (30 s), primer annealing at 56 °C (20 s) and extension for 1.5 min at 72 °C. The final extension was done at 72 °C (10 min) for 1 cycle followed by the hold at 4 °C. The second round of the nested PCR was done by following a similar protocol but 7 µl of the first-round product was used as template and final primer annealing was done at 58 °C Besides the primers for tk amplification, several internal primers were also used for the tk whole gene sequencing (Table 2).

The PCR products were analysed on 1% agarose gels containing SYBR Safe (Invitrogen, USA) for nucleic acid staining. PCR bands of the correct size were either gel purified (QIAGEN Gel Extraction Kit, Germany) or PCR purified (QIAGEN PCR Purification Kit, Germany) prior to DNA sequencing. Sequences were analysed using the MEGA^42^ and BioEdit^43^.

### Real-time PCR

A quantitative real-time PCR was developed to determine the virus DNA copy number in the DNA from the total cellular yield. A 302 bp fragment of the HSV-1 *UL5* gene was amplified using the primers *UL5*-4F and 4R (Table 2). PCR product from *UL5*-4F and 4R primer of HSV-1 was cloned into a TOPO-TA (Invitrogen) plasmid. Copy number of the construct was calculated from the plasmid concentration and molecular weight of the plasmid plus insert. Serial dilutions of this recombinant plasmid were used as known standards to calculate the standard curve during each experiment and HSV DNA copy number of the samples was calculated from this standard curve. PCR mixture contained 10 µL of SYBR Green qPCR Master Mix (Genet Bio), 0.5µL each of 0.3 mM *UL5*-4F and 4R primers, 2.5µL of standard or 5 µl test sample and RNase-free water in a final volume of 20 µL. The thermal cycling program in the Quant Studio 5 (Applied Bioscience) consisted of 5 min at 95 °C to activate the Taq DNA polymerase, followed by 40 cycles of 15 s at 95 °C, 30 s at 58 °C and 30 s at 72 °C. Fluorescence was monitored during the 72 °C extension phase.

### Immunofluorescence

For all the IF assays, 3.0 × 10^4^ cells were grown overnight onto poly-D-lysine-coated coverslips (Bluestar). The cells were infected with approximately 10^4^ copies of non-CPE viruses and 20 p.f.u. HM3PP (HSV-1 wt). After 48 h P.I., cells were fixed in 4% paraformaldehyde, permeabilized with 0.5% Triton-X-100, blocked with 1% BSA and stained with anti-ICP4 or anti-VP16 primary antibodies (Abcam), pre-labelled with Alexafluor 488 (Invitrogen). Another immunofluorescence staining was done with Anti-HSV ICP5 antibody (Abcam) pre-labelled with Alexafluor 568 (Invitrogen, USA) to see the expression of HSV late protein. After washing in 1XPBS, the cells were mounted in Prolong Antifade Diamond mounting agent with DAPI (Invitrogen, USA) and imaged (Biorad Zoe microscope or Olympus confocal microscope).

### Western blot

Western blot analysis was carried out to measure the expression of the ICP4, an early expressed HSV-1 protein and GAPDH, a cellular house-keeping protein. Along with this, expression level of capsid protein, VP16 was also checked. HSV-1-infected cell suspensions were concentrated in a small volume with Amicon 100 kD filter (MERK, CB82301) to reduce the load of cellular proteins. This was followed by lysis with lysis buffer containing 8 M urea. Cell suspensions were denatured at 95 °C for 5 min, with Laemlli buffer and electrophoresed at 90 V for 3 h through a 10% bis-acrylamide gel. The gels were blotted onto nitrocellulose membranes using Semi-Dry blot transfer apparatus (Bio-Rad) and incubated overnight. The membrane was saturated with blocking solution (5% milk) for 1 h at room temperature and then incubated overnight at 4 °C with the primary antibody diluted at 1:1000. Mouse monoclonal antibody against ICP4 (Abcam), mouse monoclonal anti-HSV VP16 (Abcam) and HRP-conjugated rabbit monoclonal antibody for GAPDH (CST) were used. After overnight incubation, the membranes were washed three times in wash buffer (1% Tween-PBS). The membranes were incubated with goat-anti mouse secondary antibody (Novus) diluted 1:12,000 for ICP4 or VP16. The membranes were further washed three times with 1XPBS and a chemiluminescent substrate (Bio-Rad) was then applied to the membranes for 1 min prior to the exposure in a Chemi-Doc (Bio-Rad).

### Cloning and SDM

Wt tk gene was amplified with H1TKCF/CR primer containing Hind-III and Eco-R1 cut site and cloned into pCDNA-EGFP3.1 plasmid to clone tk gene form wt HSV-1 DNA. The DNA was extracted from HM3PP-infected Vero cells using DNeasy Kit (QIAGEN) according to the manufacturer's instructions. The entire HSV-1 tk gene coding sequence was PCR amplified from this DNA with the forward primer H1TKCF (5′-TGCTCGAGCATGGCTTCGTACCCCTGCCATCAACACGC-3′) and the reverse primer H1TKCR (5′-GGAATTCCTCAGTTAGCCTCCCCCATCTCC-3′). These primers contained Hind-III and Eco-R1 cut sites respectively. PCRs were performed under the following conditions: one denaturation step for 5 min at 95 °C; 45 cycles of melting at 95 °C for 30 s, annealing at 56 °C for 20 s, and extension at 72 °C for 1.5 min; and a final elongation step for 10 min at 72 °C. The PCR products were purified with a PCR purification kit (QIAGEN), and then the tk gene amplicons were cloned into the plasmid. Selected clones were confirmed by sequencing. Together with forward primer H1TKCF and reverse primer H1TKCR, a set of two primers (H1TKF4, H1TKR1) spanning the entire coding region of the tk gene was utilized for DNA sequencing.

Wt tk clone was subjected to site-directed mutagenesis (SDM) to obtain the mutations, R212K or E225K in the HM3PP-tk backbone. SDM was done using QuikChange site-directed mutagenesis kit (Agilent Technologies, La Jolla, CA) following the manufacturer’s instructions (SDM primers in Table 2). Introduction of mutation(s) in HSV-1 tk clone was confirmed by sequencing.

### Acyclovir sensitivity assay

The sensitivity of the plaque-forming HSV-1 and 2 isolates to ACV was tested by plaque reduction assay (PRA) in Vero cells, as described before^44^. In brief, aliquots of the plaque-forming viruses were diluted to contain 100 p.f.u./well and inoculated into 12-well tissue culture plates (Thermo Fisher) containing approximately 2 × 10^5^ Vero cells/well. After adsorption for 60 min at 37 °C in humidified 5% CO2 incubator, a DMEM1-overlay, thickened with high-density carboxymethylcellulose (CMC) (SIGMA) and containing different concentrations of ACV, was added to each well. The plaques were stained with crystal violet solution and enumerated at 72 h P.I.

To check the ACV-sensitivity of the non-CPE HSV-1 clinical isolates, full tk gene of a non-CPE virus (MAQ) was amplified by nested PCR. A549 cells were transfected using FuGene HD (Promega, USA) with the purified PCR amplicons of full-length MAQ-tk gene and super-infected with a plaque-forming HSV-1 wt virus (HM3PP) at MOI = 3. The infected yield was harvested at 24 h P.I. and titrated in Vero cells in the presence of ACV (in CMC-overlay) at higher than IC90 concentration (i.e., 4.4 µM) to select for ACV-resistance. Plaques, when observed at higher-than-expected frequency at 72 h P.I. (≥ 10 in 10^4^ p.f.u. inoculum), were picked and passaged in Vero cells. The DNA from such plaques were analysed to confirm the successful transfer of the resistant tk gene to otherwise ACV-sensitive HSV-1 wt by homologous recombination. Resultant recombinant viruses (e.g., HM-MAQ tk-Rec), selected in presence of inhibitory drug concentration, were tested by PRA as described above.

A similar approach was taken to characterise the ACV-sensitivity of the non-CPE HSV-1 mutations, R212K and E225K. However, recombinant plaque-forming HSV-1 could not be selected like the HM-MAQ tk-Rec. So, a tk trans-complementation assay was done to indirectly determine ACV-sensitivity/resistance. Both tk mutant plasmids (generated through SDM) were transfected (1.0 µg/well) individually in 12-well plates containing A549 cells, with FuGene HD™ transfection reagent and incubated for 24 h. Then the cells were washed with 1xPBS, followed by infection with HM3PP or HM-MAQ tk-rec (tk-null virus). After 1 h adsorption, cells were topped with HD-CMC-DMEM-1 with and without 4.4 µM ACV to the individual wells. After 72 h P.I, cells were stained with crystal violet to count the plaques. Plasmid containing HM3PP wt tk gene was used as control for these complementation assays.

### AFM imaging of HSV-1

Virus-infected cells (approx. 10^6^) were disrupted by pulse sonication in a bath sonicator. The cells were then centrifuged for 5 min at 1000 g to remove the cell debris. The supernatant was collected and centrifuged again at 14,000 g for 2.5 h at 4 °C. The resultant pellet was collected and eluted in 100 µl sterile TE buffer (0.01 mol/L Tris, 0.001 mol/L ethylenediaminetetraacetic acid, pH 7.6).

As a positive control, approx. 10^7^ p.f.u. of the HSV-1 wt (HM3PP) was processed as described above. For HSV-1 wt, 50 µl of the elute was treated with 0.2% Triton-X 100 in 1:1 ratio for 30 min. A 100-fold dilution of the mix was placed on the mica sheet and observed using AFM. In the case of non-CPE HSV-1 isolate (e.g., RBG-A1), 10 µl of the sample was treated with 0.2% Triton-X 100 in 1:1 ratio for 30 min and 50-fold dilution was prepared to observe under the AFM.

50 µl of the aforesaid elute of RBG-Pa1 (passaged once in A549 cells) was immuno-precipitated using Immunoprecipitation Kit-Dynabeads™ Protein G (Invitrogen, USA), following the standard protocol suggested by the manufacturer. Monoclonal Anti-HSV VP16 antibody (Abcam) was used as the trapping antibody for immunoprecipitating the target antigen i.e., HSV tegument protein VP16.

The final eluate obtained after immune-purification was further treated with 0.2% Triton-X 100 in 1:1 ratio for 30 min to mechanically disrupt the purified virus-antibody complexes. A 15-fold dilution was prepared, followed by pulse sonication. The diluted sample was then centrifuged at 13,000 rpm for 5 min. The clear supernatant, thus obtained, was observed using AFM. Both, immunoprecipitated and non-immunoprecipitated samples were diluted in nuclease-free water, as required and 5 µl of each diluted sample was applied on freshly cleaved muscovite Ruby mica sheet (ASTMV1 grade) and allowed to air dry. Once the sample was fixed, the mica sheet was put through AFM.

## Supplementary Information

Supplementary Information.

## Data Availability

The datasets presented in this study can be found in online repositories. The names of the repository/repositories and accession numbers can be found in the article/supplementary material. The GenBank (https://www.ncbi.nlm.nih.gov/genbank/) accession numbers of the submitted sequence of HSV tk gene are provided below along with the isolate name: HSV1-PB3PP: MW582652; HSV1-HM3PP: MW582653; HSV1-AB2: MW582654; HSV1-WTB: MW582655; HSV1-ASW: MW582656; HSV1-RBG: MW582657; HSV1-SS: MW582658; HSV1-TH: MW582659; HSV1-AM: MW582660; HSV2-AKS: MW582661; HSV2-BKN: MW582662; HSV1-MAQ: MW582663.
