# Supplementary material for: Non-cytopathic herpes simplex virus type-1 isolated from acyclovir-treated patients with recurrent infections
Source: Sci Rep. 2022 Jan 25;12:1345. doi: 10.1038/s41598-022-05188-w (PMC8789845; doi:10.1038/s41598-022-05188-w)
Supplement: Supplementary file 1 — Supplementary Information. [file 41598_2022_5188_MOESM1_ESM.pdf]

SUPPLEMENTARY INFORMATION

SUPPLEMENTARY METHODS AND RESULTS

Attempts to reactivate non-cytopathic viruses in cell culture using corticosteroid

HSV-1 generally produces latency in neurons. As the non-CPE HSV-1 isolates were showing latency- like symptoms in epithelial cell lines (eg. A549), pilot level experiments were done to see whether they reactivate and produce CPE.

Thus, representative non-CPE viruses were subjected to treatment with dexamethasone (**SIGMA**) in cell culture to see whether they reactivate as a cytopathic virus. For this, A549 cells were seeded at a concentration of $5x10^4$ cells/well in 12-well plates 24hrs prior to the experiment. Cells were infected with non-CPE viruses. Copy number of the inoculum was pre-determined by qPCR. HSV-1 wild-type (wt) (HM3PP wt) was inoculated (20 p.f.u./well) as cytopathic virus control and the infected cell cultures were harvested at 48hrs P.I. Cells infected with the non-CPE viruses were incubated for 120hrs P.I. with DMEM1 containing 50nM dexamethasone. DNA was isolated from the virus-infected cell harvests with DNeasy Kit and copy number was determined by qPCR.

Results

Non-CPE viruses were cultured in the presence of dexamethasone which is known to reactivate HSV from latency [1,2]. Copy numbers of the harvested samples were compared to that of the inoculum. In presence of dexamethasone, no improvement was observed in the growth of non-CPE viruses. Rather decrease in the virus copy number was observed in both cases i.e., for cytopathic and non-CPE viruses, possibly due to reduced cell duplication in presence of steroid (data not shown).

References

1. Dreyer, L. L., Sydiskis, R. J. & Bashirelahi, N. Effect of dexamethasone on herpes simplex virus replication in mouse neuroblastoma cells (NB41 A3): Receptor characteristics. *J. Clin. Lab. Anal.* **3**, 236–243 (1989).
2. Du, T., Zhou, G. & Roizman, B. Induction of apoptosis accelerates reactivation of latent HSV-1 in ganglionic organ cultures and replication in cell cultures. *Proc. Natl. Acad. Sci. U. S. A.* **109**, 14616–14621 (2012).

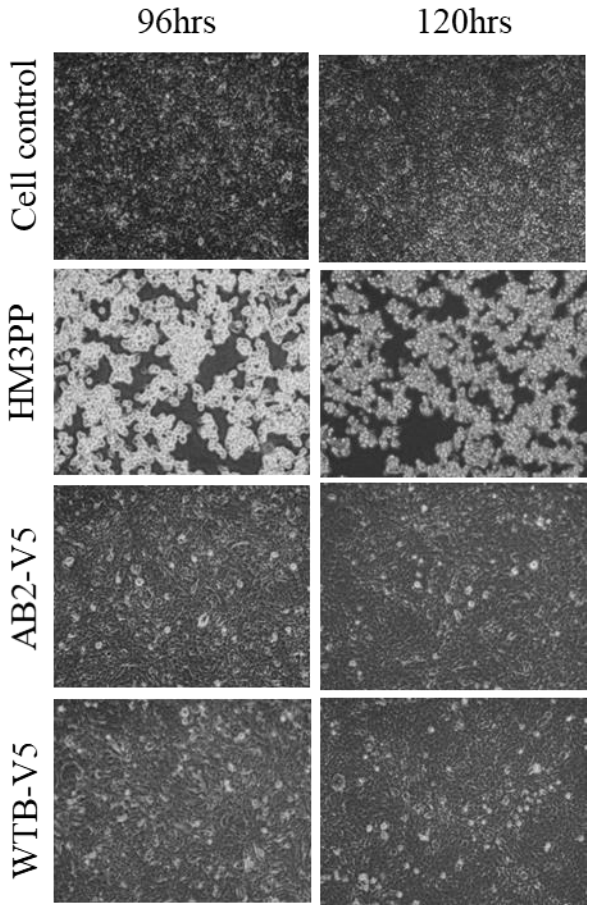

Figure S1: Infection of HSV-1 clinical isolates in Vero cell culture. The **representative** images show the individual isolates at 96hrs and 120hrs P.I., corresponding to Figure 1(b).

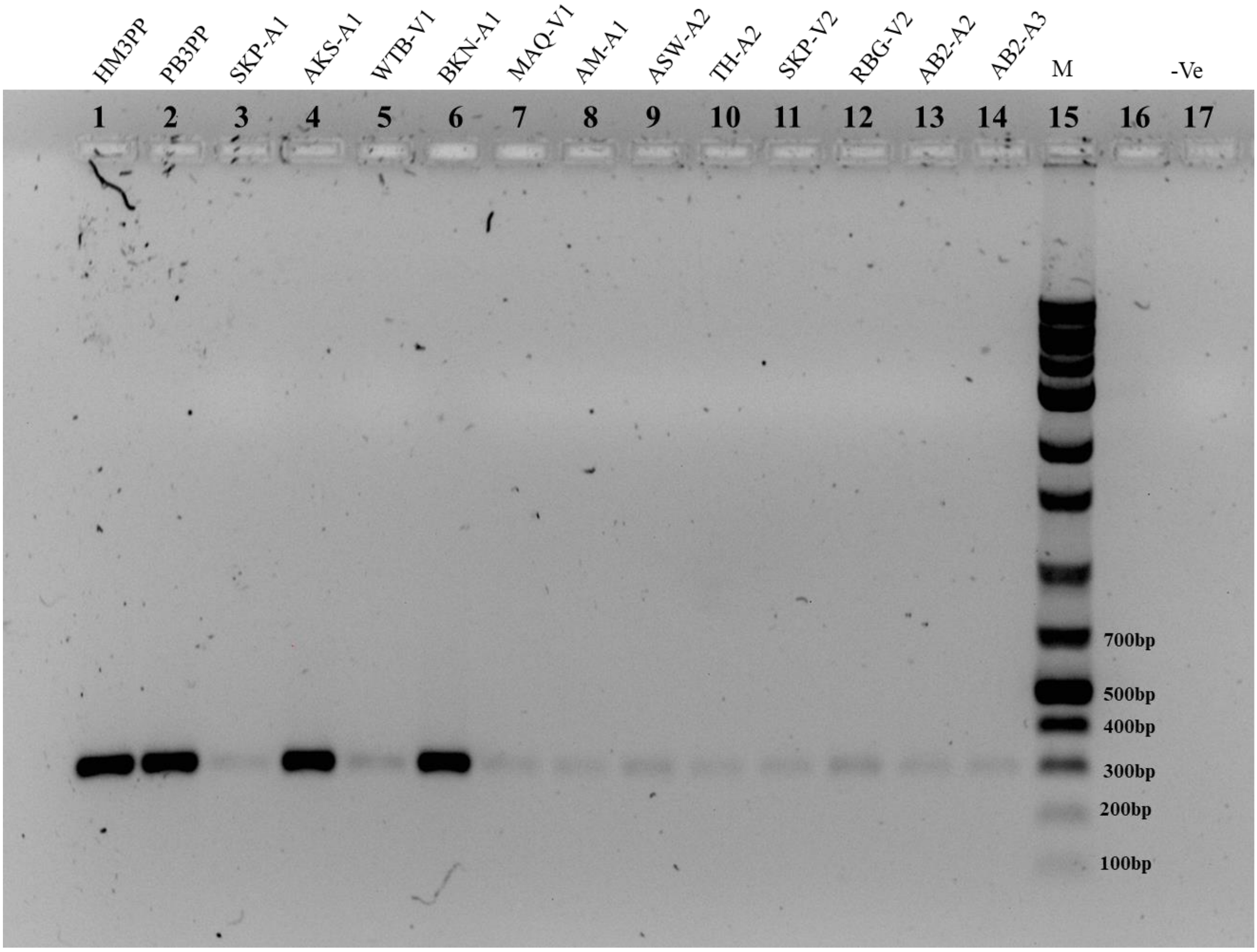

Figure S2: Representative 1% agarose gel image of PCR products, amplified from HSV *UL5* gene, confirming the presence of HSV in different passages in A549 and Vero cells. PCR amplification of *UL5* gene fragment using UL5-4F/4R primers (302 bp product). In comparison to the cytopathic viruses (lanes 1, 2, 4 and 6), the non-CPE viruses produced faint bands. After several passages these non-CPE viruses remained low-replicating, producing faint PCR bands (lanes 9-14). Samples are marked with their passage number in the respective cell lines, 'A' denotes A549 cell line and 'V' stands for Vero cell line. The adjacent number denotes the passage number for a given cell line. The molecular marker is marked as 'M'. –Ve stands for PCR negative control, i.e. PCR mix without template DNA.

Schematic plan of tk gene sequencing

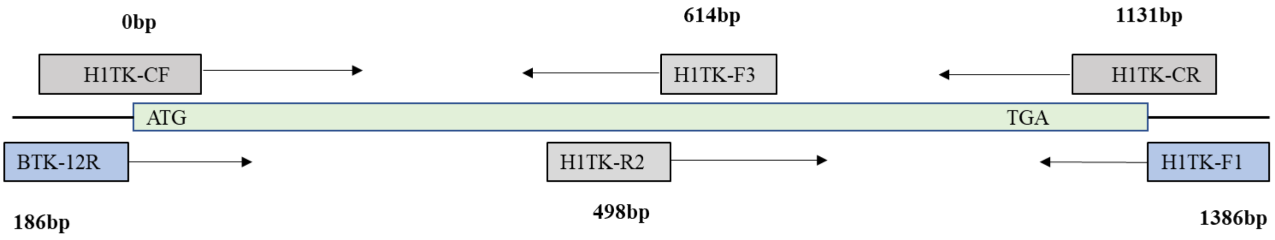

Figure S3: A schematic diagram showing the amplification and sequencing strategy for HSV tk gene. Start of the tk gene is marked as ATG. H1TK-F1 and BTK-12R were designed to amplify from the outside of the TK. Nested PCR was designed with H1TK-CF and H1TK-CR. Two internal primers, H1TK-F3 and H1TK-R2 were designed to read the nucleotides from inside (Table 2).

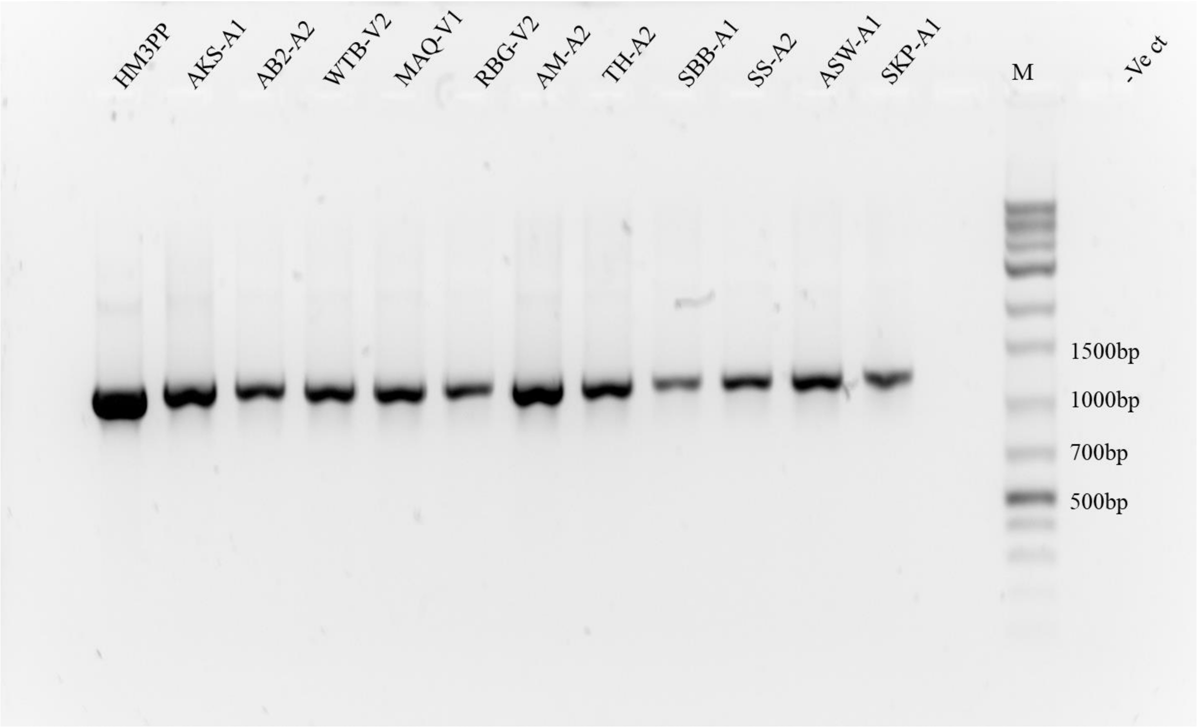

Figure S4: Representative 1% agarose gel image of HSV-1 tk gene-specific PCR products and controls. The first-round PCR was done with H1TK-F1/BTK-12R to amplify the nucleotide region from 46435 to 47819 (1385bp) (according to HSV-1 isolate KOS 1.1, accession number KT887224). Second-round nested PCR with H1TK-CF/-CR primers yielded the 1131bp band for HSV-1 positive samples, visible in the above gel. The 10-fold diluted first-round PCR products were used as the template. Samples are marked with their passage number for the respective cell line, where ‘A’ denotes A549 and ‘V’ stands for Vero. The molecular marker is marked as ‘M’. Negative control with nuclease-free water as template, was used in all PCR reactions.

(a)

```
                                      10        20        30        40        50        60        70        80        90       100
                              ....|....|....|....|....|....|....|....|....|....|....|....|....|....|....|....|....|....|....|....|
AB032881.1:HSV-1 strain h16   MASYPCHQHASAFDQAARSRGHSNRRTALRPRRQQEATEVRLEQKMPTLLRVYIDGPHGMGKTTTTQLLVALGSRDDIVYVPEPMTYWQVLGASETIANI
JQ780693.1|HSV-1 strain KOS   ....................................................................................................
LT594105.1:                   ....................................................................................................
LT594112.1:                   ....................................................................................................
LT594110.1:                   ....................................................................................................
KX265046.1:                   ....................................................................................................
KJ847330.1:IND/2011           ....................................................................................................
MH319852.1:NIV/IND/2019       ....................................................................................................
PB3PP-MW582652                ....................V...............................................................................
HM3PP-MW582653                ....................V...............................................................................
AB2_A2-MW582654               ....................................................................................................
AB2_V1                        ....................................................................................................
WTB_A2                        ....................................................................................................
WTB_V2-MW582655               ....................................................................................................
ASW_A1-MW582656               ....................................................................................................
RBG_V2-MW582657               .......................................................V................................R...........
SS_A2-MW582658                ....................................................................................................
TH_A2-MW582659                ....................................................................................................
AM-V2-MW582660                ....................................................................................................
MAQ_A1                        .......................................................A............................................
MAQ_V1-MW582663               .......................................................A............................................
HM-MAQ_tk-Rec                 .......................................................A............................................
pcDNA-EGFP3.1-HM3PP_tk_cl-10  ....................................................................................................

                                     110       120       130       140       150       160       170       180       190       200
                              ....|....|....|....|....|....|....|....|....|....|....|....|....|....|....|....|....|....|....|....|
AB032881.1:HSV-1 strain h16   YTTQHRLDQGEISAGDAAVVMTSAQITMGMPYAVTDAVLAPHIGGEAGSSHAPPALTLIFDRHPIAALLCYPAARYLMGSMTPQAVLAFVALIPPTLPG
JQ780693.1|HSV-1 strain KOS   ....................................................................................................
LT594105.1:                   ....................................................................................................
LT594112.1:                   ....................................................................................................
LT594110.1:                   ....................................................................................................
KX265046.1:                   ....................................................................................................
KJ847330.1:IND/2011           ....................................................................................................
MH319852.1:NIV/IND/2019       ....................................................................................................
PB3PP-MW582652                ....................................................................................................
HM3PP-MW582653                ....................................................................................................
AB2_A2-MW582654               ..............................................G..............N............................G.........
AB2_V1                        ..............................................G..............N............................G.........
WTB_A2                        ....................................................................................................
WTB_V2-MW582655               ....................................................................................................
ASW_A1-MW582656               ....................................................................................................
RBG_V2-MW582657               ....................................................................................................
SS_A2-MW582658                ....................................................................................................
TH_A2-MW582659                ....................................................................................................
AM-V2-MW582660                ....................................................................................................
MAQ_A1                        ....................................................................................................
MAQ_V1-MW582663               ....................................................................................................
HM-MAQ_tk-Rec                 ....................................................................................................
pcDNA-EGFP3.1-HM3PP_tk_cl-10  .............T......................................................................................

                                     210       220       230       240       250       260       270       280       290       300
                              ....|....|....|....|....|....|....|....|....|....|....|....|....|....|....|....|....|....|....|....|
AB032881.1:HSV-1 strain h16   TNIVLGALPEDRHIDRLAKRQRPGERLDLAMLAAIRRVYGLLANTVRYLQGGGSWREDWGQLSGTAVPPQGAEPQSNAGPRPHIGDTLFTLFRAPELLAP
JQ780693.1|HSV-1 strain KOS   ....................................................................................................
LT594105.1:                   ....................................................................................................
LT594112.1:                   ....................................................................................................
LT594110.1:                   ....................................................................................................
KX265046.1:                   ....................................................................................................
KJ847330.1:IND/2011           ....................................................................................................
MH319852.1:NIV/IND/2019       ....................................................................................................
PB3PP-MW582652                ....................................................................................................
HM3PP-MW582653                ....................................................................................................
AB2_A2-MW582654               ...........K............K...........................................................................
AB2_V1                        ...........K............K...........................................................................
WTB_A2                        ........................K.....................................L.....................................
WTB_V2-MW582655               ........................K.....................................L.....................................
ASW_A1-MW582656               ....................................................................................................
RBG_V2-MW582657               ....................................................................................................
SS_A2-MW582658                ....................................................................................................
TH_A2-MW582659                ....................................................................................................
AM-V2-MW582660                ........................K...........................................................................
MAQ_A1                        ....................................................................................................
MAQ_V1-MW582663               ....................................................................................................
HM-MAQ_tk-Rec                 ....................................................................................................
pcDNA-EGFP3.1-HM3PP_tk_cl-10  ....................................................................................................

                                     310       320       330       340       350       360       370
                              ....|....|....|....|....|....|....|....|....|....|....|....|....|....|....|..
AB032881.1:HSV-1 strain h16   NGDLYNVFAWALDVLAKRLRPMHVFILDYDQSPAGCRDALLQLTSGMVQTHVTTPGSIPTICDLARTFAREMGEAN*
JQ780693.1|HSV-1 strain KOS   ............................................................................*
LT594105.1:                   ...............................................I............................*
LT594112.1:                   ............................................................................*
LT594110.1:                   ............................................................................*
KX265046.1:                   ............................................................................*
KJ847330.1:IND/2011           ............................................................................*
MH319852.1:NIV/IND/2019       ............................................................................*
PB3PP-MW582652                ............................................................................*
HM3PP-MW582653                ............................................................................*
AB2_A2-MW582654               ............................................................................*
AB2_V1                        ............................................................................*
WTB_A2                        ............................................................................*
WTB_V2-MW582655               ............................................................................*
ASW_A1-MW582656               ............................................................................*
RBG_V2-MW582657               ............................................................................*
SS_A2-MW582658                ............................................................................*
TH_A2-MW582659                ............................................................................*
AM-V2-MW582660                ............................................................................*
MAQ_A1                        TATCITCLPGPWTSWPNASV.CTSLSWITTNR.PAAGTPCCK.PP.WSRPTSPPQAPYRRSATWRARLPGRW.RLT
MAQ_V1-MW582663               TATCITCLPGPWTSWPNASV.CTSLSWITTNR.PAAGTPCCK.PP.WSRPTSPPQAPYRRSATWRARLPGRW.RLT
HM-MAQ_tk-Rec                 TATCITCLPGPWTSWPNASV.CTSLSWITTNR.PAAGTPCCK.PP.WSRPTSPP?APYRRSATWRARLPGRW.RLT
pcDNA-EGFP3.1-HM3PP_tk_cl-10  ............................................................................*
```

(b)

```
Z86099.2:HSV-2(strain HG52)   MASHAGQQHAPAFGQAARASGPTDGRAASRPSHRQGASGARGDPELPTLLRVYIDGPHGVGKTTTSAQLMEALGPRDNIVYVPEPMTYWQVLGASETLTN
M29943.1 HSV-2                ......................................E.............................................................
M29941.1 HSV-2                ......................................E.............................................................
MF564037.1:HSV-2              ......................................E......................................D......................
AKS_A1- MW582661              ......................................E......................................D......................
BKN_A1- MW582662              ......................................E.............................................................

                                     110       120       130       140       150       160       170       180       190       200
                              ....|....|....|....|....|....|....|....|....|....|....|....|....|....|....|....|....|....|....|....|
Z86099.2:HSV-2(strain HG52)   IYNTQHRLDRGEISAGEAAVVMTSAQITMSTPYAATDAVLAPHIGGEAVGPQAPPALTLVFDRHPIASLLCYPAARYLMGSMTPQAVLAFVALMPPTAP
M29943.1 HSV-2                ..............................................?.....................................................
M29941.1 HSV-2                ....................................................................................................
MF564037.1:HSV-2              ....................................................................................................
AKS_A1- MW582661              ....................................................................................................
BKN_A1- MW582662              ....................................................................................................

                                     210       220       230       240       250       260       270       280       290       300
                              ....|....|....|....|....|....|....|....|....|....|....|....|....|....|....|....|....|....|....|....|
Z86099.2:HSV-2(strain HG52)   GTNLVLGVLPEAEHADRLARRQRPGERLDLAMLSAIRRVYDLLANTVRYLQRGGRWREDWGRLTGVAAATPRPDPEDGAGSLPRIEDTLFALFRVPELLA
M29943.1 HSV-2                ....................................................................................................
M29941.1 HSV-2                ......................H.............................................................................
MF564037.1:HSV-2              ....................................................................................................
AKS_A1- MW582661              ..............................................................P.....................................
BKN_A1- MW582662              ....................................................................................................

                                     310       320       330       340       350       360       370
                              ....|....|....|....|....|....|....|....|....|....|....|....|....|....|....|..
Z86099.2:HSV-2(strain HG52)   PNGDLYHIFAWVLDVLADRLLPMHLFVLDYDQSPVGCRDALLRLTAGMIPTRVTTAGSIAEIRDLARTFAREVGGV*
M29943.1 HSV-2                ............................................................................*
M29941.1 HSV-2                ............................................................................*
MF564037.1:HSV-2              ............................................................................*
AKS_A1- MW582661              ............................................................................*
BKN_A1- MW582662              ............................................................................*
```

Figure S5: Multiple sequence alignment of TK protein from (a) HSV-1 and (b) HSV-2 clinical isolates. GenBank accession numbers have been mentioned for the HSV reference sequences used in the alignments for HSV-1 (a) and HSV-2 (b). These reference sequences were identified as closest to the clinical isolates' TK sequences by BLAST search. The GenBank accession numbers of the HSV-1 and HSV-2 clinical isolates, in this study, have been mentioned against the respective isolates. TK sequence was confirmed during the passages for HSV-1. In case of the HSV-1 isolates with TK mutations/polymorphisms, the sequences from different passages revealed that the overall sequence, including the amino acid changes in TK, remained unchanged during the cell passages.

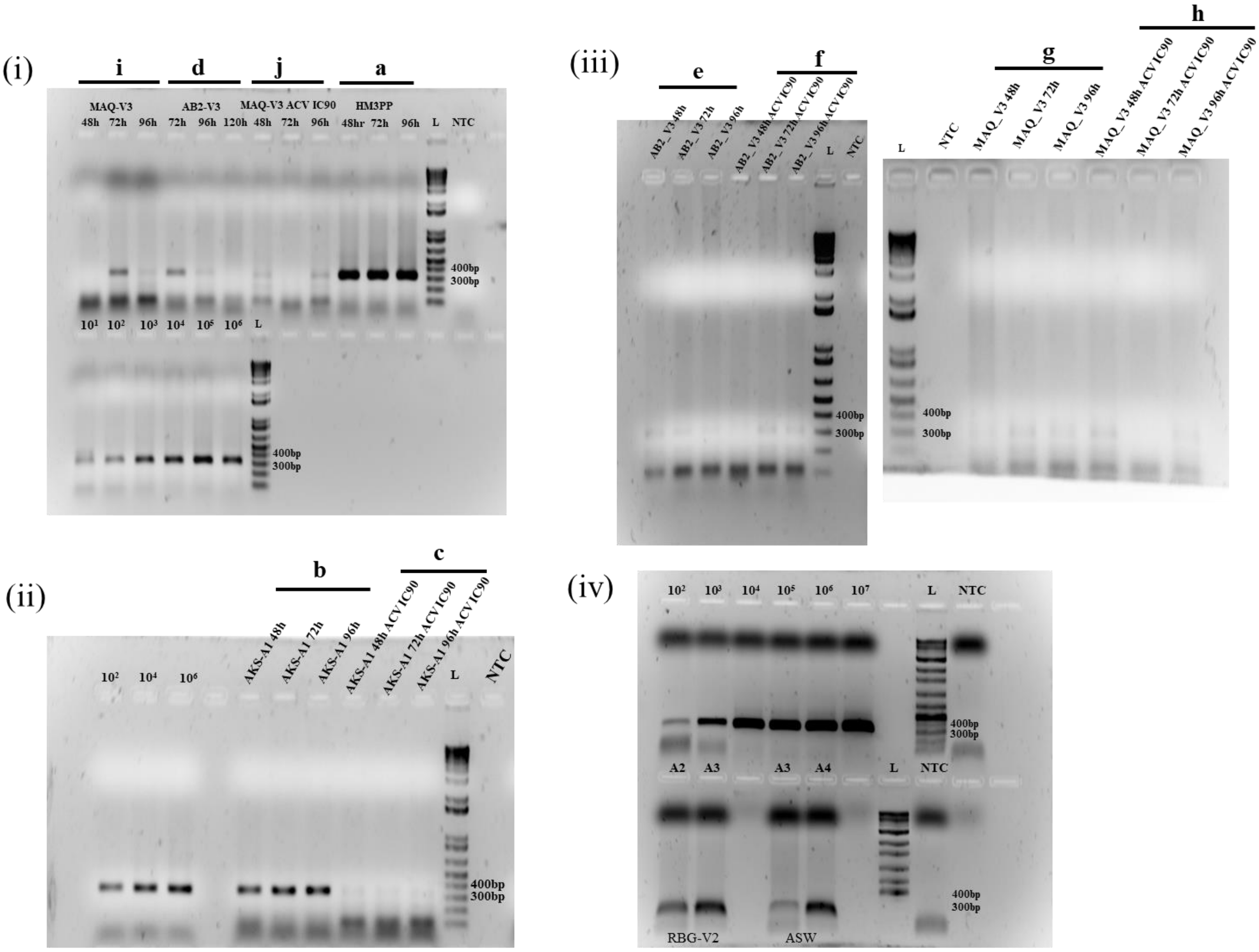

Figure S6: Agarose gel electrophoresis of the representative qPCRs (Figure 2a) to confirm specific amplification by visualization of PCR bands. A 302bp target (part of the *UL5gene*) was amplified to quantify the viruses.

Gel images **(i)-(iii)** correspond to Figure 2a. The relevant qPCR experiments are demarcated by small letter alphabets shown on top of the panel.

(iv) Gel image corresponding to Figure 2b is shown, where increment of the virus copy number was determined at different cell passages.

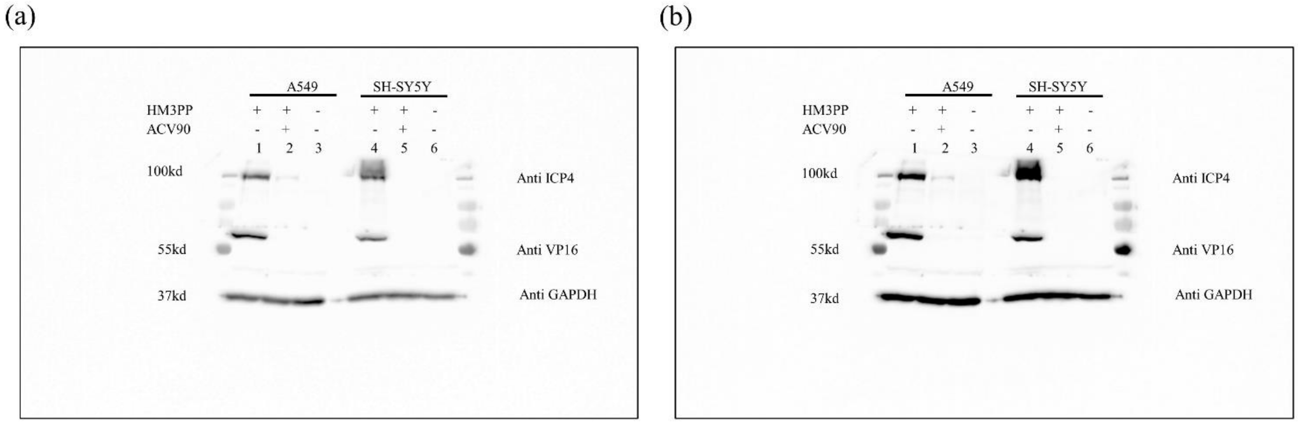

Figure S7: Western blot image showing expression of HSV-1 wt early protein, ICP4 and late protein, VP16 in epithelial and neuronal cells. (a) 2 sec exposure and (b) 10 sec exposure images of blots have been presented here in reference to **Figure 4 (a)** in the main article. Further details have been provided in the legend of **Figure 4 (a).**

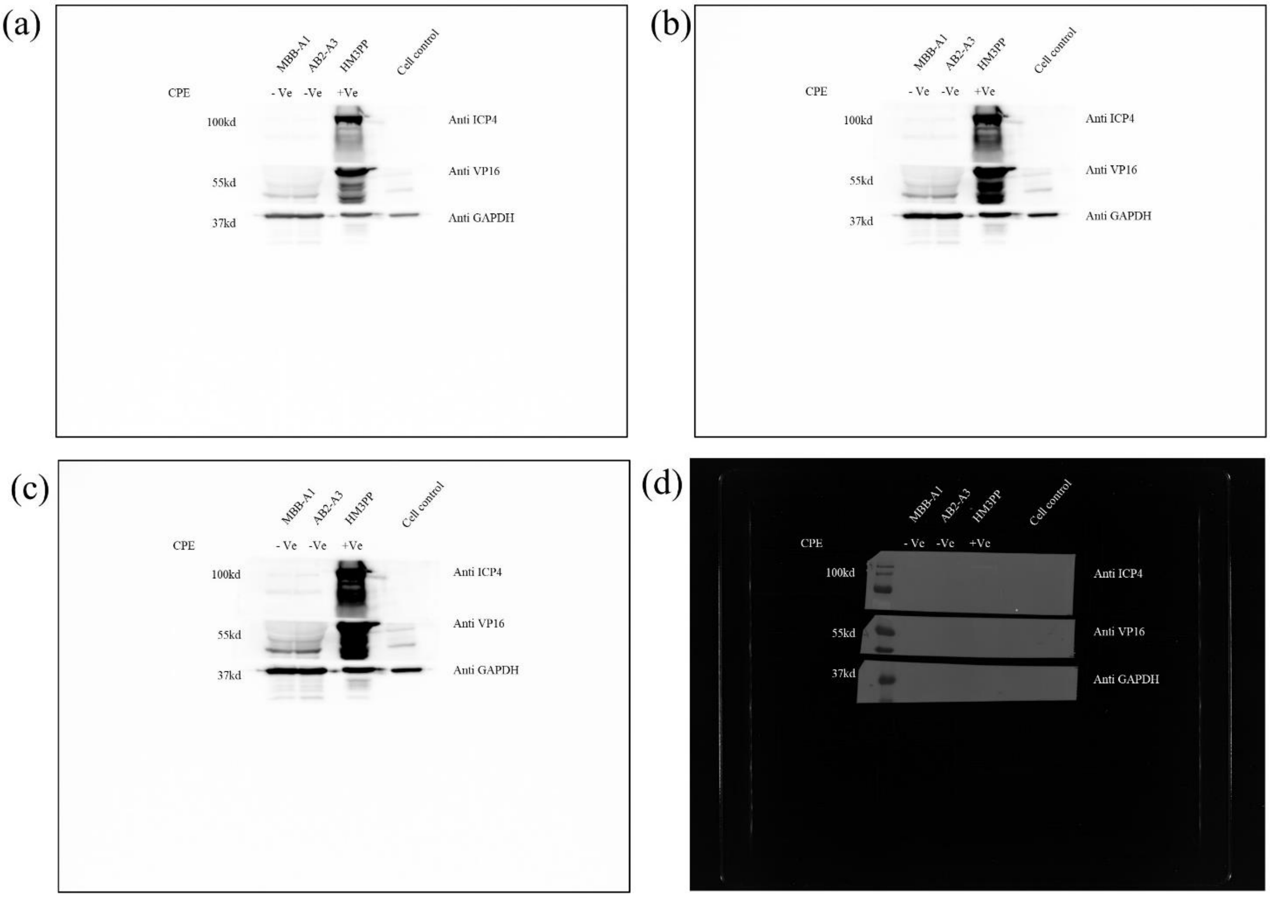

Figure S8: Western blot image showing eexpression of immediate early protein (ICP4) and late protein (VP16) in A549 cell line for two representative non-CPE viruses (MBB and AB2). (a) 2 sec exposure, (b) 5 sec exposure, (c) 10 sec exposure and (d) visible light images of the blots that were used to obtain the final image, presented in **Figure 4 (b).** The visible light image of the blots (d) was taken to align the different protein bands with the protein marker/ladder. Further details have been provided in the legend of **Figure 4 (b).**

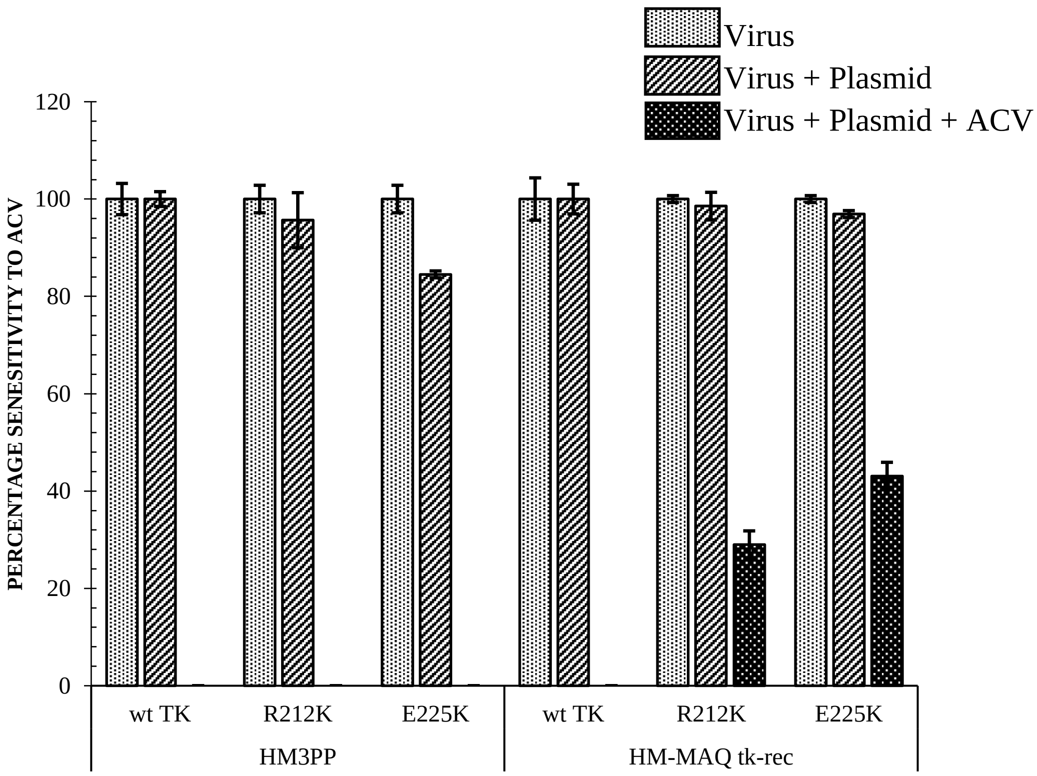

Figure S9: TK trans-complementation assay to ascertain response to ACV for novel TK mutations identified in non-cytopathic HSV-1 isolates.
A549 cells were transfected with individual tk mutant plasmids (i.e. wt-tk, R212K-tk or E225K-tk plasmid). The transfected cells were infected with HM3PP (HSV-1 wt) or ACV-resistant (HM-MAQ tk-rec) TK null virus.

In presence of 4.4µM ACV (>IC90 for HM3PP), the mutant TK-expressing wells did not show any plaque (i.e. ACV-resistance) in case of HM3PP infection. This was due to the fact that HM3PP infection resulted in wt-TK production which successfully phosphorylated ACV for effective antiviral activity (third column of second and third sets of columns from the left).

But HM-MAQ tk-rec (TK null virus) was fully resistant to >44.0µM ACV. The HM-MAQ tk-rec was found sensitive to ACV when inoculated into wt-tk plasmid-transfected cells due to supply of wt-TK in trans (third column of fourth set of columns from the left). The TK null virus could not activate ACV; however, the mutant TKs could only partially phosphorylate ACV. Thus, the R212-tk and E225K-tk-transfected wells produced 30% and 40% plaques compared to the no-drug controls respectively (third column of fifth and sixth sets of columns from the left, respectively). So, the TK null virus showed 70% and 60% ACV-resistance in presence of R212K and E225K mutant TK enzymes (supplied *in trans*) against 4.4µM ACV respectively. The columns represent mean ± SD.
